# Prioritizing disease and trait causal variants at the *TNFAIP3* locus using functional and genomic features

**DOI:** 10.1038/s41467-020-15022-4

**Published:** 2020-03-06

**Authors:** John P. Ray, Carl G. de Boer, Charles P. Fulco, Caleb A. Lareau, Masahiro Kanai, Jacob C. Ulirsch, Ryan Tewhey, Leif S. Ludwig, Steven K. Reilly, Drew T. Bergman, Jesse M. Engreitz, Robbyn Issner, Hilary K. Finucane, Eric S. Lander, Aviv Regev, Nir Hacohen

**Affiliations:** 1 Broad Institute of MIT and Harvard, Cambridge, MA 02142 USA; 2 Klarman Cell Observatory, Broad Institute of MIT and Harvard, Cambridge, MA 02142 USA; 3 Department of Systems Biology, Harvard Medical School, Boston, MA 02115 USA; 4 Program in Biological and Biomedical Sciences, Harvard Medical School, Boston, MA 02115 USA; 5 Analytic and Translational Genetics Unit, Massachusetts General Hospital, Boston, MA 02114 USA; 6 Program in Bioinformatics and Integrative Genomics, Harvard Medical School, Boston, MA 02115 USA; 7 Department of Organismic and Evolutionary Biology, Harvard University, Cambridge, MA 02138 USA; 8 Harvard Society of Fellows, Harvard University, Cambridge, MA 02138 USA; 9 Department of Biology, Massachusetts Institute of Technology, Cambridge, MA 02142 USA; 10 Howard Hughes Medical Institute, Cambridge, MA 02142 USA; 11 Center for Cancer Research, Massachusetts General Hospital, Boston, MA 02114 USA

**Keywords:** Epigenomics, Functional genomics, Immunogenetics, CRISPR-Cas9 genome editing

## Abstract

Genome-wide association studies have associated thousands of genetic variants with complex traits and diseases, but pinpointing the causal variant(s) among those in tight linkage disequilibrium with each associated variant remains a major challenge. Here, we use seven experimental assays to characterize all common variants at the multiple disease-associated *TNFAIP3* locus in five disease-relevant immune cell lines, based on a set of features related to regulatory potential. Trait/disease-associated variants are enriched among SNPs prioritized based on either: (1) residing within CRISPRi-sensitive regulatory regions, or (2) localizing in a chromatin accessible region while displaying allele-specific reporter activity. Of the 15 trait/disease-associated haplotypes at *TNFAIP3*, 9 have at least one variant meeting one or both of these criteria, 5 of which are further supported by genetic fine-mapping. Our work provides a comprehensive strategy to characterize genetic variation at important disease-associated loci, and aids in the effort to identify trait causal genetic variants.

## Abstract (web summary)

While genome-wide association studies have yielded thousands of trait-associated loci, identifying causal variants remains challenging. Here, the authors perform seven genomics assays in various cell types to prioritize genetic variants in the *TNFAIP3* locus, and report high-priority variants within disease-associated haplotypes.

## Introduction

Genome-wide association studies (GWAS) have revealed >100,000 associations of genetic variants with human traits and diseases (e.g. autoimmune disease), but it remains a challenge to pinpoint the causal variant(s) that account for the association by altering disease risk and determine their functions^1–4^. This is because they are often in tight linkage disequilibrium (LD) with non-causal variants and, in the vast majority of cases, lie in non-coding regions, where it is more challenging to predict the impact and relevant context of variants.

Most causal variants in the non-coding genome are likely to act through altering transcript abundance in a disease-relevant context. In the relevant context (cell type, tissue source, stimulation, genetic background, and disease status), experimental assays could be used to characterize the relationship between genetic variants and gene regulation. However, there are several challenges in this strategy. First, one or more aspects of the relevant context may be unknown. Second, even in the relevant context, there are many possible impacts of non-coding variants (such as different effects on gene expression or isoform usage), and each would involve a separate experimental assay, highlighting different features. Third, although ideally the relationship would be tested by allelic substitution in the relevant context—for instance, by CRISPR-directed base editing or homologous recombination^5–8^, this approach is difficult to scale at present. As a result, various assays have been proposed for identifying potentially causal variants, based on the variant’s relation to or impact on different molecular features in a relevant cell type.

These assays can be categorized into four classes, depending on (i) whether they involve observations of natural systems or engineered experimental perturbations and (ii) whether they pertain to a region or an individual variant.

1. Observational assays that characterize the genomic region in which the variant resides. Examples include using ATAC-seq, DNase-I-seq, and H3K27ac ChIP-seq^1,4,9,10^, as well as testing whether the variant lies in spatial proximity to a target gene, based on topological assays such as 4C or HiC^11,12^.
2. Observational assays that characterize the impact of naturally occurring genetic differences at the variant. Examples include characterizing whether the variant shows allele-specific association with expression of one or more nearby genes or with local chromatin features (that is, an expression quantitative trait locus (eQTL) or a chromatin QTL, respectively), or whether the variant disrupts a transcription factor (TF) motif.
3. Engineered perturbational assays that test the impact of the genomic region containing the variant. Examples include assaying the effect of CRISPR-directed inhibition (e.g., CRISPRi^13^) and activation (e.g., CRISPRa^14^) of the region on the expression of nearby genes or on chromatin organization.
4. Engineered perturbational assays that test the impact of the variant itself. Examples include testing allele-specific enhancer activities in massively parallel reporter assays (MPRAs) and related methods^15–18^.

These assays have been used in previous studies to suggest particular genetic variants as more likely to impact disease risk. However, we do not know the extent to which each of these assays actually enriches for causal variants.

Here, we reason that assays that usefully prioritize disease-causal variants could be recognized by testing whether they effectively enrich for disease-associated variants among all variants across a region. However, because disease causal variants for most associations are unknown, we use disease-associated variants (which are known and highly enriched for causal variants). As a proof of concept, we optimize and apply seven assays to characterize all known common genetic variants in the *TNFAIP3* locus, a genetic locus associated with multiple autoimmune diseases^19^, and where disease-associated genetic and epigenetic features have been studied extensively^20–24^. We use cell lines derived from T cells, B cells, and monocytes (U937 or THP-1 monocyte cell lines, GM12878 or BJAB B cell lines, or Jurkat T cell line), representing three major cell lineages that can impact autoimmunity. We find that two criteria are correlated with significant enrichment for the subset of SNPs that show disease/trait-association and, by inference, the subset of SNPs that play a causal role in these associations. These two criteria are: (i) localization within CRISPRi-sensitive regions in one of the cell types, or (ii) localization within open chromatin regions while also showing allele-specific reporter activity by MPRA. We find SNPs that fulfill at least one of these two criteria in 9 of 15 disease/trait-associated *TNFAIP3* haplotypes, prioritizing 18 putatively causal SNPs in the locus associated to 15 diseases. By contrast, several other criteria showed no enrichment for disease/trait association. Our results highlight the limitations of using individual assays for implicating a variant as potentially functional, and suggests that a combination of assays, cell types and context will be needed to prioritize variants at disease loci.

## Results

### The *TNFAIP3* locus harbors 15 independent disease associations

**Fig. 1:**
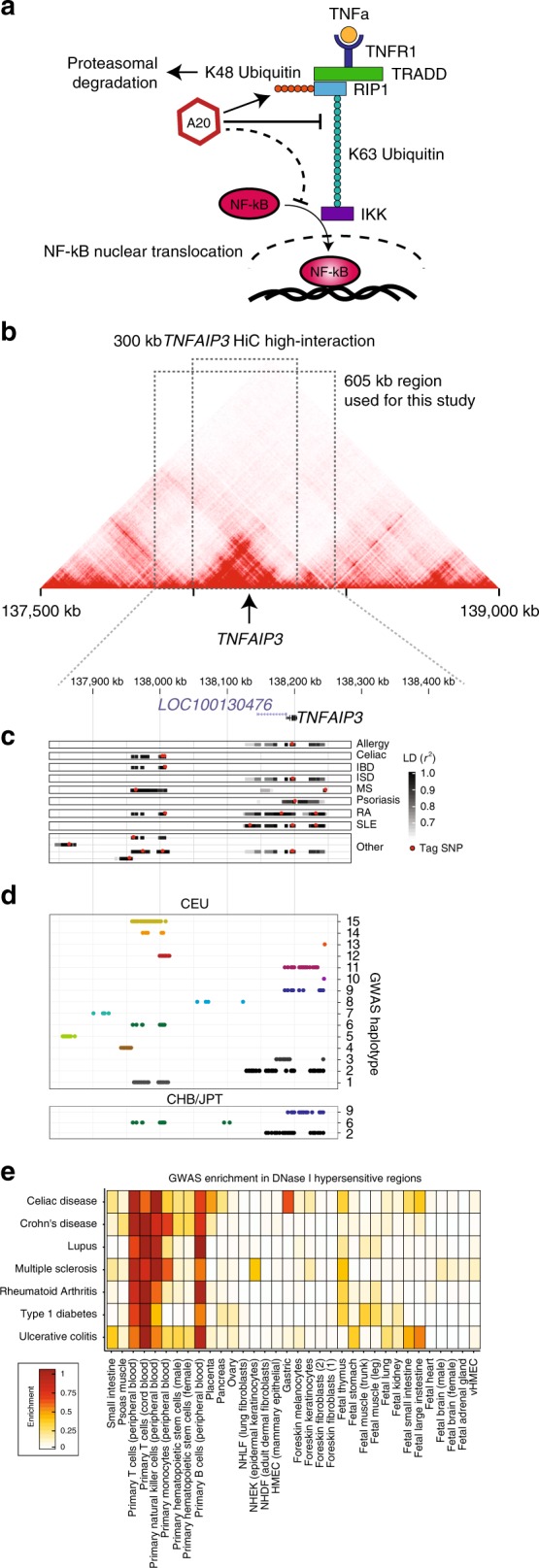
Disease variants in the complex autoimmune-associated *TNFAIP3* locus. **a** *TNFAIP3* encodes the A20 protein, which forms part of a negative feedback loop to dampen NF-kB-mediated immune activation. **b** HiC plots for the lymphoblastoid B cell line GM12878, with color intensity proportional to the interaction frequency between genomic coordinates (*x-*axis). Boxes indicate the 300 kb high-interaction domain and the 605 kb region used in this study. **c**, **d** Genetics of the *TNFAIP3* locus. The positions (shared *x*-axis indicated above **c**) of variants with respect to the *TNFAIP3* gene and a lncRNA (LOC100130476). **c** GWAS tag SNPs (red) and SNPs in tight LD (greyscale boxes indicating LD to tag SNP) for many immune-related phenotypes (*y*-axis). **d** GWAS haplotypes defined by combining all SNPs in tight LD (*r*^2^ > 0.8) to GWAS tag SNPs for European (CEU; top) and East Asian (CHB/JPT; bottom) populations. Colors are used to help identify shared haplotypes between CEU and CHB/JPT populations. **e** Autoimmune GWAS signals are enriched in open chromatin of immune cells. Heritability enrichment (color) of disease-associated SNPs in DHS of various tissues (*x*-axis) for seven autoimmune diseases (*y*-axis), according to LD-score regression. Also see Supplementary Data 1, 2.

As a test case, we investigated the *TNFAIP3* locus because it has strong associations to many autoimmune diseases. *TNFAIP3* encodes the A20 protein, which is upregulated by NF-kB upon immune stimulation, and dampens pathways that activate NF-kB in a negative feedback loop (Fig. 1a)^19,25,26^. At least 49 GWASs have identified genome-wide significant SNPs in the *TNFAIP3* locus that together are associated with 16 human diseases and phenotypes, including lupus (SLE), rheumatoid arthritis (RA), psoriasis, inflammatory skin disorder (ISD), celiac disease, inflammatory bowel disease (IBD), and multiple sclerosis (MS). Rather than focusing only on disease-associated SNPs (that is, those showing genome-wide-significant associations for one of these diseases as tag SNPs or in tight LD to them), we systematically examined all common SNPs (MAF > 0.01) in the ~300 kb topologically associating domain (TAD) containing *TNFAIP3* (based on HiC data from GM12878 B cells and THP-1 monocyte cell lines^12,27^), and 150 kb on either side of the TAD because it is known that regulatory regions can affect the expression of genes outside of TADs^28^ (Fig. 1b, top; Supplementary Fig. 1). We reasoned that studying all common non-coding variants would allow us to derive empirical null distributions for each assay because most variants are not expected to be functional. Accordingly, we selected for analysis all 2776 common variants with minor allele frequency > 0.01 in East Asian or European populations (in 1000 Genomes, see “Methods” section).

We next analyzed the locus to estimate the number of SNPs that contribute to disease. Of the 2776 variants, 294 were in tight LD (*r*^2^ > 0.8) to at least one of 34 ‘tag SNPs’—that is, a SNP reported as having the highest association score in one of the GWASs for the autoimmune and other diseases noted above (Fig. 1c**;** Supplementary Fig. 2a). Through LD analysis (*r*^2^ ≥ 0.8) of the tag SNPs, we identified 15 independent haplotypes associated with one or more GWAS traits in Europeans (Fig. 1d**;** Supplementary Fig. 2b–d); three of these haplotypes also overlapped East Asian disease-associated haplotypes, but with slight differences in the associated SNPs (Fig. 1d**;** Supplementary Fig. 2d). Notably, fine-mapping of immune-related UK Biobank phenotypes (autoimmune disease (self-reported or diagnosed), self-reported allergy, and eosinophil counts) showed that, despite limited sample size, all but two of these separately fine-mapped alleles were contained on three of the 15 disease-associated haplotypes from our LD analysis (Supplementary Data 1, 2, see “Methods” section). Collectively, we estimate that at least 15 SNPs in the locus contribute to disease.

While *TNFAIP3* is likely to play a role in many disease-relevant cell types, we chose to study T cells, B cells, and monocytes. These important innate and adaptive immune cell types likely play a role in the autoimmune diseases with which the *TNFAIP3* locus is associated because their localization in disease-associated tissues, signaling, and function are correlated with disease progression in the clinic and in animal models of disease^29–34^. T cell-, B cell-, and monocyte-specific accessible chromatin and active histone marks (H3k27ac and H3K4me3 ChIP-seq) are also significantly enriched (compared to other cell types) for GWAS variants (*P* < 1 × 10^−8^) from studies of diseases that had associations in *TNFAIP3* according to stratified LD score regression^35^ (Fig. 1e; Supplementary Fig. 3a–c). Moreover, deleting *TNFAIP3* in these cell types causes systemic autoimmunity in mice^36–40^.

We studied cell lines derived from these cell types: THP-1 and U937 for monocytes, BJAB and GM12878 for B cells, and Jurkat for T cells. The chromatin accessibility profiles of these cell lines are enriched for autoimmune-associated risk variants similarly to the corresponding primary cells (Supplementary Fig. 3d), and among blood cell types profiled by ATAC-seq^20^ they were most similar to the cell type they represent (Supplementary Fig. 4a), especially at the TNFAIP3 locus (Supplementary Fig. 4b), suggesting that the selected cell lines could serve as models for these cell types.

### A panel of assays to annotate genetic variation

**Fig. 2:**
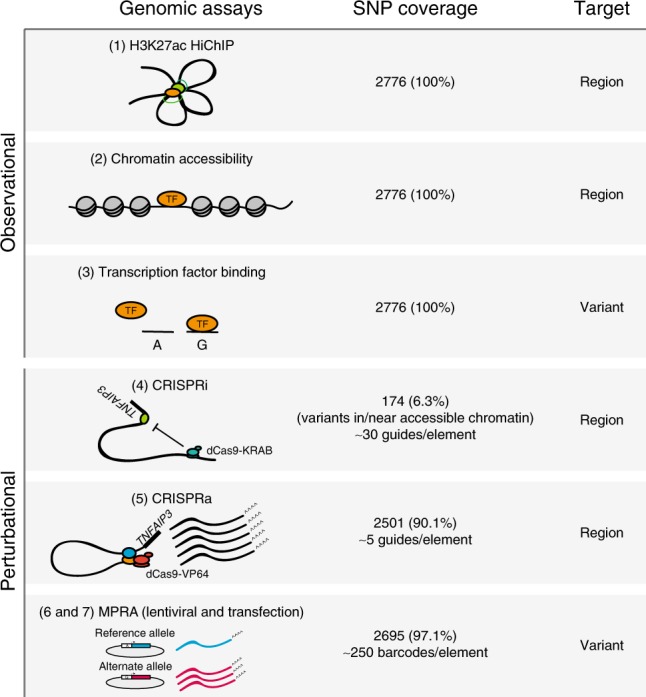
Seven approaches for characterizing non-coding genetic variants. Genomic assays (left), the coverage of all common genetic variants in the 605 kb locus (middle), and whether the assay is specific to genomic regions or variants (right), grouped into observational (top) and perturbational assays (bottom). (1) HiChIP can be used to identify active chromatin regions (H3K27ac labeled) that interact with the *TNFAIP3* promoter. (2) DHS and ATAC-seq can be used to identify regions of accessible chromatin. (3) Variants predicted to alter TF binding can be identified using motif analysis in combination with evidence of TF binding by ChIP-seq^42^. Also see Supplementary Data 3–9. (4 and 5) Pooled CRISPRi and CRISPRa screens can determine regulatory potential of each region by repressing (CRISPRi) or artificially inducing (CRISPRa) each targeted region. (6 and 7) MPRA (with lentiviral or transfection delivery strategies) can be used to test for allele-specific reporter expression.

We used both observational and perturbational assays to characterize regulatory features in the regions where variants were located, and the variants themselves (Fig. 2).

Using observational assays, we first analyzed regions that contact the *TNFAIP3* promoter (primary T cell and GM12878 B cell HiChIP data; ~5 kb resolution^41^) and regions of accessible chromatin in any of the cells lines (using ATAC-seq in unstimulated and stimulated cells (Supplementary Fig. 5a, b), and publicly available DHS of cell types from the blood^42^). For each variant, we also assessed whether it lies within a region bound by a TF based on ChIP-seq^42^, and whether the variant is predicted to affect TF binding according to its cognate motif (Supplementary Fig. 5c).

Using perturbational assays, we sought to identify regions that can affect *TNFAIP3* expression. With CRISPRi (in which KRAB-dCas9 binds to a region targeted by a guide RNA and represses chromatin locally^13^), we identified regions whose inhibition alters *TNFAIP3* expression. We targeted all regions with accessible chromatin in either U937, BJAB, or Jurkat cell lines, tiled guides across each element (and up to 100 bp on either side), and identified guides and regions that significantly repress *TNFAIP3* expression (see the “Methods” section; Supplementary Fig. 6, Supplementary Data 4–6). We also applied CRISPRa (which relies on dCas9-VP64 with MS2 stem loops that recruit HSF1 and p65 to artificially activate gene expression^14^), using guides that target 50 bp regions surrounding each variant in the *TNFAIP3* locus to identify regions with the potential to induce *TNFAIP3* expression (Supplementary Fig. 7, Supplementary Data 5–7). For shared guides and regions, we confirmed that CRISPRi and CRISPRa drove the expected opposing changes in expression of *TNFAIP3* (Supplementary Fig. 7d, e). We also tested for allele-specific reporter expression induced by individual variants using MPRAs. We synthesized all alleles for each variant, centered in 150 bp of the surrounding reference DNA. These were cloned upstream of the *TNFAIP3* promoter driving the expression of a GFP gene that contained sequence barcodes in the 3′ UTR. We used these barcodes to read out expression of each allele by RNA-seq. We delivered them to immune cell lines by either lentivirus (L-MPRA) to integrate them into chromosomes, or transfection (T-MPRA) to generate extrachromosomal reporters (Fig. 2; Supplementary Fig. 8, Supplementary Data 8, 9). Variant-driven expression of the reporter was reproducible within, but not between, the two delivery methods (Supplementary Fig. 8b).

For each assay, we determined which SNPs scored as ‘hits’ based on SNPs being within regions annotated as: (i) interacting with the *TNFAIP3* promoter by HiChIP; (ii) accessible by ATAC-seq/DHS; (iii) within a region that modulates *TNFAIP3* expression based on CRISPRi/CRISPRa; or (iv) displaying allele-specific reporter activity using MPRA (see the “Methods” section).

### Hits from two strategies enrich for disease-associated SNPs

**Fig. 3:**
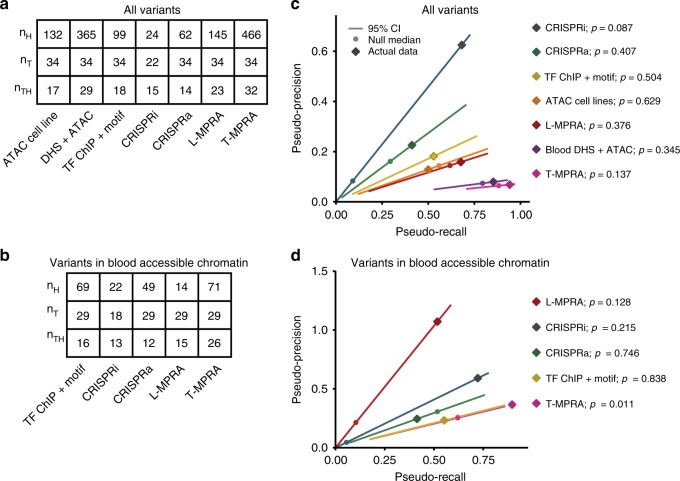
Comparison of GWAS enrichment across methods. **a**, **b** Values for *n*_*H*_, *n*_*T*_, and *n*_*TH*_ for all methods, considering (**a**) all variants, and (**b**) only variants in open chromatin. **c**, **d** Pseudo-precision (*y* axes) and pseudo-recall (*x* axes) for GWAS enrichment for each assay (colors), with diamonds depicting the actual assay performance (as in Supplementary Fig. 9a), and the lines depicting the 95% CI of each assay’s null distribution (as in Supplementary Fig. 9b). Empirical one-sided *P*-values derived from the genomic-shifts null are indicated next to each assay label. *P*-values are not corrected for multiple hypothesis testing. **c** Each assay evaluated individually for all tested variants and **d** considering only SNPs in blood cell accessible chromatin. The relationship between pseudo-precision and pseudo-recall is linear in the null (pseudo-precision = (*n*_*T*_/*n*_*H*_) × pseudo-recall) because both are proportional to *n*_*TH*_ and *n*_*T*_ and *n*_*H*_ are constant.

Ideally, we would assess each assay by directly testing how well it enriches for causal variants among the full set of variants assayed. However, using metrics like ‘precision’ and ‘recall’ would require that the causal variants be known with certainty. Because they are not, we instead tested how well the methods enrich for variants in tight LD with the tag SNP (as these variants are in turn enriched for true causal variants), calculating a ‘pseudo-precision’ and ‘pseudo-recall’. For each assay, we therefore quantified (1) the number of tested SNPs considered ‘hits’ in the assay (*n*_*H*_), (2) the number of tag SNPs for which at least one SNP in tight LD was tested in the assay (*n*_*T*_; i.e. recoverable tag SNPs), and (3) the number of tag SNPs for which at least one SNP in tight LD was considered an assay hit (*n*_*TH*_; i.e. recovered tag SNPs) (Supplementary Fig. 9a). We next calculated the pseudo-precision and pseudo-recall for GWAS variants for each assay. Here, we define ‘pseudo-precision’ as *n*_*TH*_/*n*_*H*_, representing the fraction of all SNPs considered hits that are recovered tag SNPs, and ‘pseudo-recall’ as *n*_*TH*_/*n*_*T*_, representing the fraction of tag SNPs that are recovered by being in tight LD with one or more hits. These terms are similar to precision and recall except that a single causal SNP might underlie multiple tag SNPs (by being in tight LD to each of them), making a pseudo-precision above 1 possible. By these measures, a highly effective approach would recover all tag SNPs (pseudo-recall = 1) with as few SNP hits as possible (high pseudo-precision). In the calculation of pseudo-precision and pseudo-recall, we did not consider GWAS tag SNPs that had no assayed variants in tight LD with that tag SNP (including the tag SNP itself) in order to not falsely penalize the assays for technical failures (e.g., lack of PAM site for CRISPR or poor coverage in MPRA). We conducted these analyses for all variants and for the subset of variants that lie in accessible chromatin in one of the three blood cell types studied (because GWAS variants are enriched in accessible chromatin^1,4^ and accessibility data is readily available for many cell types) (Fig. 3a, b).

To determine whether the pseudo-precision/pseudo-recall performance of each method is better than expected by chance, we created an empirical null distribution by randomly permuting the hit status among the assayed SNPs (1000 permutations) or by shifting the hit status of each SNP to the next adjacent assayed SNP (Supplementary Fig. 9b, c). The shift approach preserves positional clustering of hits inherent to LD and to some of the assays (e.g. CRISPRi, open chromatin). This reduces inflation of positive hits within the null that may occur by permutation, where the permuted hits may be in LD with many more tag SNPs than are possible given the clustered nature of the assay (thus increasing pseudo-precision and pseudo-recalls) (see the “Methods” section). Both shifting and permutation yielded similar results for SNPs in tight LD with GWAS tag SNPs (Fig. 3c, Supplementary Fig. 9d). For each method, we compared the pseudo-precision and pseudo-recall of actual data to the null distribution. We did this both for all variants (Fig. 3c) and the variants located in accessible chromatin in the three blood cell types (Fig. 3d).

Relative to all variants, most of the methods (ATAC-seq on our cell lines, Blood DHS + ATAC-seq on our cell lines, TF ChIP + motif, L- and T-MPRA, and CRISPRa) did not show a significant enrichment for GWAS variants (Fig. 3c). However, CRISPRi showed 7.5-fold enrichment for GWAS variants (95% C.I., [0.9375; ∞]), albeit not significant (*P* = 0.087, empirical *P*-value with genomic-shifts null) (Fig. 3a, c, Supplementary Fig. 9a).

After restricting our analysis to variants located in accessible chromatin in the three blood cell types, several of the methods (CRISPRa and TF ChIP + motif) again showed no significant enrichment for GWAS variants. However, T-MPRA showed significant enrichment (*P* = 0.011, empirical *P*-value with genomic-shifts null; 1.44-fold enrichment for GWAS, 95% CI [1.04; 5.2]; Fig. 3d, Supplementary Fig. 9e).

Both L-MPRA and T-MPRA showed greatly increased pseudo-precision with only marginally reduced pseudo-recall when restricting attention only to variants in accessible chromatin (Fig. 3d, Supplementary Figs. 9e and 10). This may be because many variants have the capacity to alter expression when tested in an enhancer assay (such as MPRA), but do not reside in a region of accessible chromatin in the relevant cell types and thus do not alter disease risk. Although L-MPRA performed well for variants in accessible chromatin, having the highest pseudo-precision of any assay, there was limited power to evaluate L-MPRA because only four variants (in tight LD to 15 tag SNPs) out of the 19 L-MPRA hits were in accessible chromatin (*P* = 0.128, empirical *P*-value with genomic-shifts null; Fig. 3d).

For CRISPRi, pseudo-precision and pseudo-recalls changed little when focusing only on variants in accessible chromatin (Fig. 3a–d, Supplementary Fig. 10), but pseudo-precision was less significant (*P* = 0.215, empirical *P*-value with genomic-shifts null) because some of the SNPs tested lay just outside (within 100 bp) regions of accessible chromatin (Fig. 3c, d, Supplementary Fig. 9d, e).

We also considered another alternative proxy for causal variants, using credible sets from fine-mapping studies (Supplementary Data 10), determining, in this case, the number of credible sets (*n*_*T*'_) that were recovered (*n*_*TH*'_) by containing one or more assay hits (*n*_*H*_). Although the SNPs in a credible set are more likely to be causal than when doing LD expansion, the limited availability of fine-mapping data restricted this analysis and reduced our statistical power. We calculated the pseudo-precision and pseudo-recall for GWAS variants for each assay in an analogous way (Supplementary Fig. 9f–k). The rates from the credible set-based analysis generally showed similar trends to the tag SNP approach, but were less significant due to the reduced sample size (Fig. 3c, d vs. Supplementary Fig. 9d–k); in addition, pseudo-precision was necessarily reduced for fine mapping due to reduced number of association signals, but with no change in assays hits.

### Prioritization of variants in disease-associated haplotypes

**Fig. 4:**
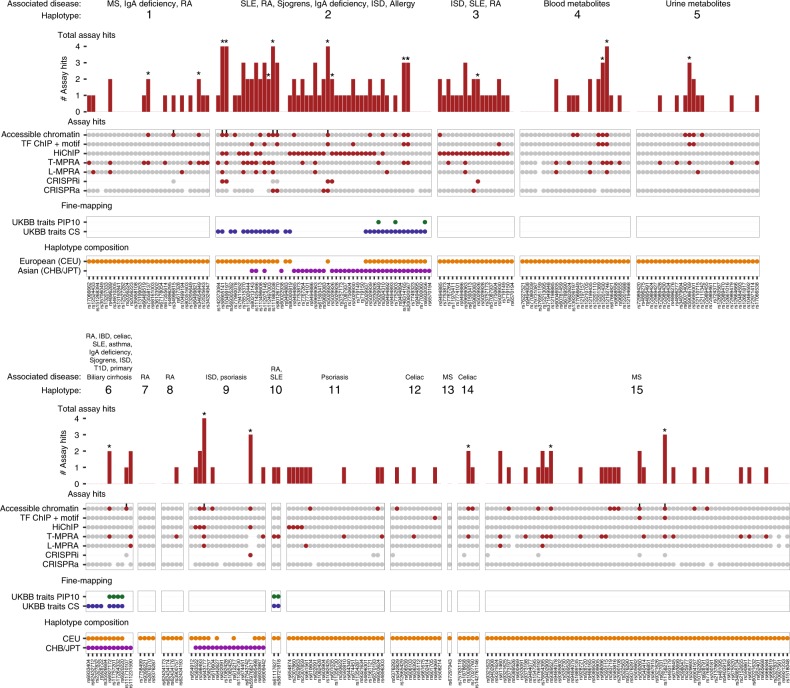
Prioritizing variants on disease-associated haplotypes. A summary of assay results and genetics data for all SNPs on each disease-associated haplotype. Each subpanel represents a different haplotype, with associated traits and the haplotype number are indicated on the top. For each SNP (*x* axes), the total number of assay hits is shown in the bar graph (top) with SNPs that are hits in CRISPRi or T-MPRA hits in accessible chromatin marked with an asterisk. Results from each assay are shown in the middle, with hits in red, and SNPs that are assayed but were not hits in gray for each of the seven assays (*y-*axis). The vertical black bars above accessible chromatin SNP status indicate SNPs that were in accessible chromatin in our tested cell lines. Fine-mapped immune-related traits from UK Biobank (UKBB), including SNPs in the 95% credible set (CS—blue) and those that have a posterior inclusion probability > 10% (PIP10—green) are second from the bottom. The population-specific SNPs contained within each disease-associated haplotype are indicated (bottom) with orange for European (CEU) and purple for East Asian (CHB/JPT). Also see Supplementary Data 3.

Finally, we used our analysis of genomic assays to prioritize SNPs on each disease-associated haplotype (Fig. 4, Supplementary Data 3). We annotated as high-priority those variants that were hits in at least one of the two assays with the best performance (CRISPRi for all variants and T-MPRA variants in accessible chromatin), finding a total of 18 such high-priority variants (Fig. 4, asterisks). Of the 15 disease-associated haplotypes, nine included one or more of these 18 SNPs. These included five SNPs that had been fine-mapped in the UK Biobank, lying in 95% credible sets representing associations with allergy, all autoimmune diseases combined, and eosinophil counts (Fig. 4, Table 1).

**Table 1:** Disease-associated variants positive for CRISPRi or chromatin accessibility with T-MPRA.

SNP ID	Associated trait	Tehranchi asATAC	Tehranchi asChIP	Fine mapped UKBB 95% CS (SuSiE)	Other evidence	Haplotype	Hit in assays
rs200820567	Allergy, ISD, RA, SLE, eosinophil counts, IgA deficiency, Sjogren’s	x		Eosinophil counts (PIP = 0.03); Allergy (PIP = 0.04)	Fine mapped in Adrianto et al. (SLE)	2	T-MPRA + accessible chromatin
rs148314165	Allergy, ISD, RA, SLE, eosinophil counts, IgA deficiency, Sjogren’s				Fine mapped in Adrianto et al. (SLE)	2	T-MPRA + accessible chromatin
rs112497003	Allergy, ISD, RA, SLE, eosinophil counts, IgA deficiency, Sjogren’s			Eosinophil counts (PIP = 0.01)		2	T-MPRA + accessible chromatin
rs111883038	Allergy, ISD, RA, SLE, eosinophil counts, IgA deficiency, Sjogren’s			Eosinophil counts (PIP = 0.01)		2	L-MPRA + accessible chromatin; T-MPRA + accessible chromatin
rs6927172	Celiac, IBD, RA, Asthma, IgA deficiency, Sjogren’s, ISD, T1D, primary biliary cirrhosis	x	NF-kB, JunD	Combined Autoimmune (PIP = 0.13)	Fine mapped in Huang et al. (UC, PIP = 0.06); Farh et al. (RA, PIP = 0.11; Celiac, PIP = 0.19; UC, PIP = 0.23); Westra et al. (RA, PIP = 0.10)	6	T-MPRA + accessible chromatin
rs643177	ISD, psoriasis		Pou2f1		Fine mapped in Farh et al. (Psoriasis, PIP = 0.15)	9	L-MPRA + accessible chromatin; T-MPRA + accessible chromatin
rs59086769	Urine metabolites					5	T-MPRA + accessible chromatin
rs1002658	Celiac	x	NF-kB, PU.1			14	T-MPRA + accessible chromatin
rs11758213	MS	x	JunD		Fine mapped in Huang et al. (UC, PIP = 0.075)	15	T-MPRA + accessible chromatin
rs9389527	MS					15	T-MPRA + accessible chromatin
rs12201430	Blood metabolites	x				4	T-MPRA + accessible chromatin
rs12192746	Blood metabolites					4	L-MPRA + accessible chromatin; T-MPRA + accessible chromatin
rs34654849	MS, IgA deficiency, RA					1	T-MPRA + accessible chromatin
rs73558137	MS, IgA deficiency, RA					1	T-MPRA + accessible chromatin
rs5029924	Allergy, ISD, RA, SLE, eosinophil counts, IgA deficiency, Sjogren’s				BJAB asATAC and fine mapped in Farh et al. (SLE, PIP = 0.09)	2	CRISPRi
rs5029926	Allergy, ISD, RA, SLE, eosinophil counts, IgA deficiency, Sjogren’s					2, 3	CRISPRi
rs10499197	Allergy, ISD, RA, SLE, eosinophil counts, IgA deficiency, Sjogren’s					2	T-MPRA + accessible chromatin; CRISPRi
rs58905141	Allergy, ISD, RA, SLE, Eosinophil Counts, IgA deficiency, Sjogren’s			Eosinophil counts (PIP = 0.02)		2	L-MPRA + accessible chromatin; CRISPRi
rs559766217	ISD, Psoriasis					9	CRISPRi

Variants that are positive for either chromatin accessibility with T-MPRA or CRISPRi are listed with their associated trait, and whether they were also positive in Tehranchi et al. as having allele-specific ATAC (asATAC) or asChIP-seq for TFs in LCLs. Our fine-mapping data using UKBB traits for the 95% credible set variants are included, and other fine-mapping data or evidence for SNP functionality is listed in Other Evidence. The haplotype for the SNP is listed in Haplotype.

Several of these high-priority variants had other evidence supporting a role in disease. For example, rs6927172 is the only high-priority variant on haplotype 6 (which lay in accessible chromatin and scored in the T-MPRA assay, but not in the CRISPRi assay); this variant is associated with many diseases, including RA, SLE, celiac, T1D, and asthma, and it is a fine-mapped SNP in our analysis of combined autoimmune disease in the UK Biobank and in previously reported studies of ulcerative colitis, RA, and celiac)^1^ (posterior inclusion probability (PIP) = 0.13^43^; Table 1, Supplementary Data 2). This variant also has evidence of allele-specific ATAC-seq and allele-specific ChIP-seq for the TFs NF-kB and JunD in lymphoblastoid cell lines^44,45^ and allele-specific ATAC-seq and allele-specific ChIP-seq for the NF-kB1 p50 subunit in primary CD4 T cells^20^. It appears to interact with the *TNFAIP3* promoter by 3C, has allele-specific reporter activity according to a luciferase assay, and lays in a region that affected *TNFAIP3* expression based on 11–12 bp CRISPR-induced deletions^46,47^. Only two of the other 10 variants on the haplotype had evidence of impact (with rs111710107 only being in accessible chromatin, and rs111231590 having allele-specific reporter activity according to both T- and L-MPRA assays).

Similarly, rs643177 is one of two high-priority variants on haplotype 9 (laying in accessible chromatin and a hit in T-MPRA assay, but not tested in CRISPRi due to the lack of a suitable guide-RNAs). This variant also had evidence of interaction with the *TNFAIP3* promoter according to HiChIP, and had allele-specific reporter expression in L-MPRAs. rs643177 is a fine-mapped psoriasis SNP^1^ and has evidence of allele-specific binding of the TF Pou2f1 (Table 1). The other high-priority variant on haplotype 9 is rs559766217, which was a hit in the CRISPRi assay, is in accessible chromatin and contacts the *TNFAIP3* promoter according to HiChIP. Four of the 17 other SNPs on the haplotype have some evidence of impact (including rs538522 and rs598493, which interact with the *TNFAIP3* promoter according to HiChIP; rs598493 and rs610604, which are located in accessible chromatin; and rs6909442, which has allele-specific reporter expression according the T-MPRA assay).

Other examples include rs11758213 on haplotype 15, which is in the 95% credible set for ulcerative colitis (PIP = 0.0074)^48^ and had evidence of allele-specific ATAC-seq and ChIP-seq for the TF JunD in LCLs^44,45^ and rs1002658 on haplotype 14, which was associated with celiac disease and had evidence of allele-specific ATAC-seq and ChIP-seq for the TFs NF-kB and PU.1^44,45^. Interestingly, haplotype 2 had 41 of 51 SNPs that scored as hits in at least one of the seven assays, including five SNPs in accessible chromatin that score as hits in the T-MPRA assay and three SNPs that scored as hits in the CRISPRi assay (Table 1).

## Discussion

GWASs effectively narrow down the search for causal variants to a small set of candidates, but determining which of the candidates contributes to disease risk remains a challenge. Because disease-causal variants are likely to be correlated with functionally relevant genomic features in the cell types in which they act, it should be possible to use genomic features to help inform the search for disease-associated variants—provided that the relevant cell types are known and can be studied (which remains a serious limitation).

To study the potential utility of various genomic features for prioritizing non-coding variants, we studied seven genomic assays in three disease-relevant cell types to assess to the extent to which they enrich for disease-associated variants within a set of 2776 common non-coding SNPs in the *TNFAIP3* locus. We found significant enrichment among high-scoring SNPs for two methods: (1) variants present in CRISPRi-responsive regulatory regions and (2) variants present in accessible chromatin that also showed allele-specific reporter activity by T-MPRA. These two criteria identified 18 *TNFAIP3* variants associated with 15 diseases on 9 haplotypes; potential functional roles for these variants in immunity were supported by additional published data (such as allele-specific ATAC-seq, ChIP-seq, and genetic fine-mapping). By contrast, the other genomic features did not provide significant enrichment.

Our data support two prioritization schemes (CRISPRi and accessible chromatin with T-MPRA) as viable methods for enriching for causal variants in the *TNFAIP3* locus. However, since perturbational methods (e.g. CRISPRi, MPRA) cannot currently be scaled to the same level as observational methods (ATAC-seq, ChIP-seq, HiChIP, and TF motif analysis), we could not test the generalizability of our findings to additional disease-associated loci, variants, and cell types.

Our focus on the *TNFAIP3* locus helps to clarify a complex region with many genetic associations through analysis of variant features and functions in three main immune cell types. While our data corroborated two reported putatively causal variants associated with lupus (rs200820567 and rs148314165 on haplotype 2), it highlighted six other variants (rs58905141, rs10499197, rs5029924, rs5029926, rs112497003, and rs111883038) on the same haplotype that are also putatively causal. Whether these variants act in concert to confer risk at this haplotype needs to be examined. While we found prioritized variants for nine haplotypes, none were found for another six haplotypes, which could be explained by lack of assay sensitivity or the variants being biologically active in other cell types and conditions. Interestingly, many haplotypes contained associations to different diseases, affirming that different autoimmune diseases could have similar autoimmune genetic etiology because they are presumably promoting disease through the same causal genetic variants^1^. Our data help explain the immense genetic complexity of the locus by prioritizing 18 of the 293 disease-associated variants, although there may be even more disease-causal variants to be found in different contexts.

Our study of common variants in the *TNFAIP3* locus provides a strategy to help guide future variant characterization studies at other loci. Increasingly accurate approaches to identify causal variants will require the development and integrated analysis of experimental methods that assess variant function.

## Methods

### Cell culture and stimulation of immune cells

BJAB (DSMZ, cat. no. ACC 757), Jurkat, Clone E6-1 (ATCC, cat. no. TIB-152), U937 (ATCC, cat. no. CRL-1593.2), THP-1 (ATCC, cat. no. TIB-202), and GM12878 (Coriell, cat. no. GM12878 LCL from B-Lymphocyte) cell lines were cultured using RPMI 1640 (ThermoFisher, 21870092) containing 10% fetal bovine serum (FBS, VWR, 97068-091; 20% for GM12878) with 1% Penn/strep (VWR, 45000-652), 1% l-glutamine (ThermoFisher, 25030081), and 1% HEPES (Sigma, H0887-100ML). Cells were maintained at a culture density between 100K and 1M cells/mL. Jurkat T cells were stimulated with 2.5 μg/mL of anti-CD3 (Biolegend, 317304) and 10 ng/mL of PMA (Sigma, P1585-1MG) for 1 h prior to harvesting for CRISPRi and MPRA, and 1 and 4 h for ATAC-seq experiments. BJAB and GM12878 B cells were stimulated with 2.5 μg/mL of anti-IgM (Sigma-Aldrich, 86620270) and 2 μg/mL anti-CD40 (ThermoFisher, 14-0409-82) for 2 h for CRISPRi and MPRA, and 1 and 4 h for ATAC-seq and 4C (BJAB) experiments. THP-1 and U937 monocytes were stimulated with 100 ng/mL LPS (Invivogen, tlrl-peklps) for 2 h for CRISPRi and MPRA, and 1 and 4 h for ATAC-seq and 4C (U937) experiments.

### Lentivirus preparation

HEK293T cells were grown using DMEM (VWR, 45000-316) with 10% FBS, 1% Penn/Strep, 1% l-glutamine, 1% HEPES (10DMEM). Cells were passaged at 80% confluence for each passage. To make lentivirus, media was aspirated from the adherent cells and Trypsin EDTA 0.25% (VWR, 45000-664) was used to create a single-cell suspension; the cells were kept at 37 °C for 4 min with Trypsin, and 10DMEM was added to a final concentration of 80%. The cells were pipetted up and down until they were in a single cell suspension. They were then counted and plated in a six-well plate at 500K cells/well in 2 mL 10DMEM. The next day, when the cells were ~70% confluent, they were transfected. pVSV-G (0.1 μg; Addgene, 8454), pPAX2 (1 μg; Addgene, 12260), and the donor plasmid (1 μg), were added to 125 μL of OPTI-MEM and mixed. 6 μL of the TransIT-LT1 (Mirus Bio, MIR 2300) transfection reagent was added to a separate tube of 125 μL OPTI-MEM (ThermoFisher, 31985062) and mixed. The OPTI-MEM LT-1 mixture was then added to the OPTI-MEM plasmid mixture, mixed, and incubated at RT for 15 min. The mixture was then added dropwise to the well. The plate was then swirled in order to ensure distribution of the mixture and effective transfection. The cells were put at 37 °C to incubate overnight and the media was changed at 24 h post-transfection, this time using 10DMEM with 1% BSA (Sigma, A7979). The cells were then incubated at 37 °C for 16 h, and the supernatant was harvested. The viral supernatant was spun at 500 × *g* for 5 min to separate cellular debris, and stored at 4 °C for up to 3 months.

### 1000 Genomes Project and GWAS catalog

We centered our study on the 2776 variants that lie within and 150 kb to either side of the *TNFAIP3* TAD, yielding a 605 kb locus (MAF > 0.01, combined CHB+JPT and CEU populations from phase 3 of the 1000 genomes project (http://www.internationalgenome.org/)). We used tabix (0.2.5) (tabix -h ftp://ftp.1000genomes.ebi.ac.uk/vol1/ftp/release/20130502/supporting/vcf_with_sample_level_annotation/ALL.chr6.phase3_shapeit2_mvncall_integrated_v5_extra_anno.20130502.genotypes.vcf.gz 6:137846078-138453052 > TNFAIP3.vcf)^49^ and vcftools (0.1.15) and Plink (v1.90b3d; vcftools --vcfg TNFAIP3.vcf --keep CEU_names.txt --out CEU --plink; plink --file CEU --out CEU; plink --bfile CEU --maf 0.01 --geno 0.01 --hwe 0.01 --out CEU.filtered --make-bed)^50,51^ to extract all alleles in the *TNFAIP3* locus (chr6:137846078-138453052, hg19) that were MAF ≥ 0.01 from the 1000 genomes phase 3 database CEU and the combination of CHB and JPT populations. For trait-associated variants, we reanalyzed GWAS summary statistics (www.immunobase.org and refs. ^52,53^) for tag SNPs and those in tight LD in 1KG samples, according to the same population in which the study was conducted (*r*^2^ > 0.8; 294 SNPs)^54^.

GWAS haplotypes were defined on the basis of tight LD between GWAS tag SNPs and other genomic SNPs. We calculated LD between GWAS tag SNPs and other SNPs using Plink (v1.90b3d; --r2 inter-chr --ld-window-r2 0.2) for both East Asian (EAS) and European (CEU) populations using 1000 Genomes data^54^. Each GWAS tag SNP and all SNPs in tight LD (*r*^2^ > 0.8) within the GWAS population of study defined our initial haplotype estimates. Any of these haplotypes that shared SNPs in tight LD (*r*^2^ > 0.8) were then merged into a single haplotype until none showed any overlap, yielding 15 haplotypes associated with one or more diseases. We found that the number of haplotypes identified was robust to this cutoff between 0.76 and 0.89. Haplotypes identified in EAS and CEU that had any overlap between the GWAS Tag SNPs were merged into a single haplotype, with population-specific membership indicated in Fig. 1 and Supplementary Fig. 2, and Supplementary Data 1. We used phased 1000 Genomes genotypes to ensure each haplotype exists at >0.5% in each population.

### Genetic fine-mapping

We performed genetic association and fine-mapping in up to 361,194 unrelated, white British individuals from the UK Biobank^55^, as determined by the PCA-based sample selection criteria (https://github.com/Nealelab/UK_Biobank_GWAS/blob/master/ukb31063_eur_selection.R). We restricted to all imputed variants with MAF > 0.01% (except for missense and protein-truncating variants annotated by VEP^56^, MAF > 0.0001%), Hardy–Weinberg equilibrium *P*-value > 1 × 10^-10^, and imputation quality (INFO) > 0.8 (https://github.com/Nealelab/UK_Biobank_GWAS). To perform association tests for binary phenotypes, we used a generalized linear-mixed model as implemented in SAIGE^57^ v0.29.4 with the minimum minor allele count (MAC) threshold, MAC > 10 for each GWAS. To perform association tests for quantitative phenotypes, we used a linear-mixed model as implemented in BOLT-LMM^58^ v2.3.2 with default settings. Phenotypes for combined autoimmune disease were derived as previously defined^58^, allergy status was self-reported, and eosinophil counts were inverse rank-based normal transformed. We included sex, age, age^2^, sex × age, sex × age^2^, and top 20 principal components as covariates. Genetic fine-mapping was performed using FINEMAP v1.3^59,60^ and the summary statistics version of susieR^43^ v0.7.1 with the maximum number of causal variants specified as 10. LD matrices were calculated from imputed dosages for individuals included in each GWAS using LDstore^61^ v2.0b. Individual variant posterior inclusion probabilities and conditional 95% credible sets are reported.

### GWAS immune cell enrichments

Heritability enrichments of traits (Fig. 1**;** Supplementary Fig. 3) in cell lines and cell types were estimated using stratified LD-score regression (s-LDSC) over accessible chromatin or histone modifications in specific cell types as previously reported^35^ by interpreting the cell type-specific repression coefficient in s-LDSC model. For hematopoietic cell types and cell lines, common variants overlapping accessibility peaks from ATAC-seq data for 13 primary cell types^62^ were used to compute the heritability enrichment. For broad tissue enrichments, DNase Hypersensitivity peaks and H3K27ac and H3K4me1 ChIP-seq peaks were overlapped with common variants to compute heritability enrichments. The −log10 *P*-values for the s-LDSC regression terms for each specific annotation were shown as a measure of enrichment.

### HiChIP data and analysis

H3K27ac HiChIP data previously generated^41^ were downloaded in.fastq format from GEO accession “GSE101498”. Biological and technical replicates of Th17, Naïve T-cell, and GM12878 H3K27ac samples were pooled and aligned with Hi-C Pro^63^. Virtual 4C plots (Supplementary Fig. 5) using a resolution of 2.5 kb and a rolling mean of 2.5 windows^41^. Per-fragment estimates of interaction strength to the *TNFAIP3* promoter were generated using hichipper^64^ and normalizing to the total number of unique fragments in each library. We used a normalized interaction score of 20 to annotate regions as *TNFAIP3* interacting.

### ATAC-seq

We used the FAST-ATAC protocol^62^. 10,000–20,000 cells were sorted into RPMI 1640 containing 10% fetal bovine serum. The cells were centrifuged at 500 × *g* for 5 min at 4 °C. All of the supernatant was aspirated, ensuring that the pellet was not disturbed. The pellet was then resuspended in the tagmentation reaction mix (25 μL 2X TD Buffer (Illumina, 15027866), 2.5 μL TD Enzyme (Illumina, 15038061), 0.5 μL 1% Digitonin (Promega, G9441), 22 μL H_2_O) and mixed at 300 RPMs at 37 °C for 30 min on an Eppendorf Thermomixer. Immediately after the incubation, samples were purified using a minElute kit (Qiagen, 28006), eluting in 10 μL. The entire sample was PCRed (a 50 μL reaction with 25 μL NEBNext, 2.5 μL F+R custom nextera primers (10 μM each; Supplementary Data 11), 10 μL of tagmented DNA, and 12.5 μL H_2_O) for five cycles with the following program (72 °C, 5 min; 98 °C, 30 s; five cycles of 98 °C, 15 s, 63 °C, 15 s, 72 °C, 1 min). We performed qPCR with 5 μL of the sample to determine the number of additional cycles required, while the rest remained on ice. The 5 μl of sample was added to a qPCR mix (5 μL of PCR, 5 μl of NEBNext, 0.5 μL F+R custom nextera primers, 0.09 μL of 100X SYBR (Invitrogen, S7563), 4.41 μL H_2_O) and qPCRed (98 °C, 30 s; 20 cycles of 98 °C, 15 s, 63 °C, 15 s, 72 °C, 1 min). The number of cycles that it took to reach 1/3 the maximum fluorescence threshold in the qPCR was then applied via PCR to the original PCR sample. Libraries were cleaned using 1.5X Agencourt XP beads and ethanol washes per manufacturer’s protocol. The DNA concentration of the sample was measured using Qubit and the average fragment size was determined using a TapeStation. Samples were then multiplexed and sequenced using 50 bp paired end chemistry at an average read-count of 30M reads per sample.

Paired-end ATAC-seq reads were mapped to the genome (hg19) using Bowtie2 (2.2.1; parameters: --maxins 2000), with duplicate reads removed using Picard (2.20.6; MarkDuplicates REMOVE_DUPLICATES=true), and peaks (clusters of reads representing open chromatin regions) called using Homer (4.6; findPeaks -style dnase).

We calculated the ATAC-seq similarity between our cell lines and primary immune cell types^20^ (Supplementary Fig. 4). We used pyatac (version 0.3.4) to get read counts for each region previously identified as having been accessible in one or more immune cell types, for GM12878^65^, Jurkat, BJAB, and U937. Pearson’s correlation coefficient was calculated comparing the log ATAC-seq counts (+0.5) per region to quantify the similarity between each of the primary immune cells as well as the other cell lines, for each profiled cell line. These were sorted in decreasing order and the top five for each cell line are displayed in Supplementary Fig. 4.

### CRISPR screens

The guide libraries targeting the TNFAIP3 locus for CRISPRi and CRISPRa are available in Supplementary Data 4 and 7. To design the guide library, all possible 20 bp sgRNAs with the Cas9 protospacer adjacent motif NGG within the region surrounding TNFAIP3 (chr6:13784700–138453100, hg19) were considered. On-target scores for each guide were determined using the Rule Set 2 method described in ref. ^66^. To determine the number of off-target locations, bowtie (0.12.7)^67^ was used to map guides to the human reference (hg19) with a maximum 10,000 matches, with up to three mismatches (parameters: -n 3 -l 15 -e 10000 -y --all -S). Using this set of potential mapping locations in the genome, off-target score was calculated using the method of Hsu et al.^68^. Briefly, single off targets were calculated as *e* moves over positional mismatches between guide and off-target, where the *m* is as below and *d* is mean pairwise distance between mismatches:

$$\mathop {{\Pi}}\limits_{e \in M} (1 - W[e]) \times \frac{1}{{\left( {\frac{{(19 - \bar d)}}{{19}} \times 4 + 1} \right)}} \times \frac{1}{{n_{{\mathrm{{mm}}}}^2}}$$

$$\begin{array}{l}M = \left[ {0,\;0,\;0.014,\;0,\;0,\;0.395,\;0.317,\;0,\;0.389,\;0.079,\;0.445,\;0.508,\;0.613,\;} \right.\\ \left. {{\hskip -73pt}0.851,0.732,\;0.828,\;0.615,\;0.804,\;0.685,\;0.583} \right]\end{array}$$

Individual off-targets are aggregated into a single guide using:

$$S_{{\mathrm{{guide}}}} = \frac{{100}}{{100 + \mathop {\sum }\nolimits_{i = 1}^{n_{{\mathrm{{mm}}}}} S_{{\mathrm{{hit}}}}(hi)}}$$

On-target scores range from 0 to 100, with 100 being optimal. Off-target scores range from 0 to 100 with 100 being no off-target effects predicted. CRISPRi guides were selected to target the locations of ATAC-seq peaks from Jurkat, BJAB, or U937—with or without stimulation (overlapping peaks merged), and aimed to tile the region uniformly, with an average of ~30 guides per element. For CRISPRa, the targeted elements were the locations of SNPs (±25 bp) and guides were selected to get ~5 guides per SNP; most SNPs with at least one guide (2501/2776). In both cases, we excluded guides for which there were off-target matches near the *TNFAIP3* locus, as well as any that had more than three off-target perfect matches anywhere in the genome. We included 770 non-targeting guides in CRISPRa library and 6282 in the CRISPRi library, which were created by reversing (but not complementing) selected targeting guides. Prior to synthesis, Gs were added to all sgRNAs not starting with a G to aid in transcription efficiency. The sgOPTI vector (for CRISPRi; Addgene, 71409) or the sgSAM vector (for CRISPRa; made in house, available upon request) was digested with BsmbI (NEB, R0580S) overnight, PCR cleaned, and the digest was repeated for two hours with thermostable alkaline phosphatase (Promega, M9910) added during the final hour of digestion. The cut vector was then gel purified using a 0.7% agarose gel. Guides for CRISPRi and CRISPRa (Supplementary Data 4, 7) were synthesized using Agilent Technologies 100K arrays, with common PCR priming sequences on each element. The oligos were amplified to add Gibson assembly homology arms, and inserted into the sgOPTI vector using Gibson assembly using 500 ng of vector and 70 ng of insert. Lentivirus (protocol in methods above) was then made for all guide libraries and CRISPR-associated vectors (see below). Stable CRISPRi-expressing GM12878, BJAB, Jurkat, U937, and THP-1 cell lines and CRISPRa-expressing BJAB, Jurkat, U937, and THP-1 were made through lentiviral transduction of these cells with a doxycycline-inducible transactivator (ClonTech, 631363) and the TRE-dCas9-KRAB-BFP construct (for CRISPRi; Addgene, 85449) or pMS2-p65-HSF1 (Addgene, 73795) and dCas9-VP64-GFP (for CRISPRa; Addgene, 61422); for both, guide libraries were infected at an MOI < 0.3, and puromycin selected for 4 days. Cells containing libraries were maintained in culture without doxycycline and used for each replicate. For each replicate, cells were split and given doxycycline 24–48 h prior to harvesting, and stimulated with relevant ligands 1–2 h prior to harvesting.

We performed FlowFISH screens^69^. For PrimeFlow experiments, 5 million cells were aliquoted in PBS in polypropylene tubes and centrifuged at 500 × *g* for 5 min. All but 100 μL of the supernatant was discarded (this step is true for every centrifugation step in this protocol) and the cells were resuspended in the residual volume. Cells were then fixed according to manufacturer protocol (ThermoFisher, 88-18005-210) using Fixation Buffer 1 for 30 min at 2–8 °C with rotating. Cells were then centrifuged at 800 × *g* for 5 min. and the supernatant was discarded. Cells were then permeabilized according to manufacturer protocol with addition of RNase inhibitors through inversion, and centrifugation at 800 × *g* for 5 min, then the supernatant was discarded. This step was repeated. A second fixation step was carried out using Fixation Buffer 2 according to manufacturer protocol, the samples were mixed, and inverted for one hour in the dark at RT. The cells were then centrifuged at 800 × *g* for 5 min at RT, and the samples were washed twice with PrimeFlow RNA Wash Buffer, centrifuging the samples at 800 × *g* between each wash for 5 min. The *TNFAIP3* target probe (ThermoFisher, VA1-20723) was added at 1X in PrimeFlow RNA Target Probe Diluent, mixed thoroughly by pipetting up and down (100 μL of probe/sample), and incubated at 40 °C for 2 h, with inversion every 30 min. 1 mL of PrimeFlow RNA Wash Buffer was added to each sample, the samples were inverted to mix, and centrifuged at 800 × *g* for 5 min, and the supernatant was aspirated. Samples were then washed with 1 mL PrimeFlow RNA Wash Buffer containing RNase inhibitors twice followed by centrifugation at 800 × *g* for 5 min. 100 μL of PrimeFlow RNA PreAmp Mix was then added to each sample and briefly vortex to mix, and the samples were then incubated for 1.5 h at 40 °C with mild vortexing once every 30 min. Samples were washed three times with 1 mL of PrimeFlow RNA Wash Buffer, and they were centrifuged at 800 × *g* for 5 min, and the supernatant was aspirated. 100 μL of PrimeFlow RNA Amp Mix was then added to each sample, the samples were mixed by votexing, and were incubated for 1.5 h at 40 °C with mild vortexing once every 30 min. The cells were then washed twice in 1 mL of PrimeFlow RNA Wash Buffer and centrifuged at 800 × *g* for 5 min. Each sample received 100 μL of PrimeFlow RNA Label Probe diluted in PrimeFlow RNA Label Probe Diluent and incubated for 1 h at 40 °C with mild vortexing once at 30 min. Samples were then washed with 1 mL of PrimeFlow RNA Wash Buffer at RT followed by centrifugation at 800 × *g* for 5 min. The samples were then washed five times with 35 °C PrimeFlow RNA Wash Buffer following each wash with centrifugation at 800 × *g* for 5 min. Samples were then left in 100 μL of PBS and stored in the dark at 4 °C until sorting.

Cells expressing CRISPRi or CRISPRa constructs along with sgRNA libraries were sorted into six 10% bins, sorting on the extremes of expression (30% on either the low or high portion of the expression distribution, each divided into three contiguous bins each comprised of ~10% of the overall distribution). For each experiment and cell type, between 300K and 1M cells were sorted per bin. Genomic DNA for each sample was then reverse-crosslinked using ChIP Lysis Buffer (1% SDS, 0.01 M EDTA, 0.05 M Tris–HCl pH 7.5). Briefly, sorted cells were spun at 800 × *g* for 10 min at 4 °C, the supernatant was aspirated, and the cells were resuspended in 50 μL of ChIP Lysis Buffer, and incubated at 65 °C for 10 min. The samples were then cooled to 37 °C and 2 μL of RNase Cocktail (ThermoFisher, AM2286) was added to each sample and the sample was mixed well by pipetting, followed by incubation at 37 °C for 30 min. 10 μL of Proteinase K (NEB, P8107S) was added to each sample and the sample was mixed well by pipetting, followed by incubation at 37 °C for 2 h and then 95 °C for 20 min. gDNA was extracted using Agencourt XP beads at 0.7X following the manufacturers protocol, and the sample was eluted at 100 μL. Libraries were prepared by PCR of each sample, splitting each into four 50 μL reactions (25 μL NEB Next Master Mix, 2.5 μL barcoded sequencing forward and reverse primers (Supplementary Data 11), 11.5 μL gDNA, and 11.5 μL ddH_2_O; program: 98 °C for 30 s, 25 cycles of 98 °C for 15 s, 62 °C for 15 s, 72 °C for 16 s, then 72 °C for 2 min. The libraries were then gel purified using a 2% gel (expected band size of 206 bp). Samples were sequenced aiming to get >1,000,000 reads per bin, on either an Illumina HiSeq 2500 or MiSeq using a custom sequencing and index primers for CRISPRi and CRISPRa (Supplementary Data 11).

For CRISPRi/a analysis, reads covering the guide sequences from each bin were aligned to the designed guide sequences using Bowtie2 (2.2.1; default settings)^70^, and the total number of each guide observed in each bin counted. Read counts from each bin were modeled as if originating from a negative binomial distribution, where the underlying distribution of cells targeted by each guide had a log(expression level) that was normally distributed for each guide, with the same variance as the entire distribution (since most guides are expected to have no effect) and different means (that varied based on the effect of the guide). The percent of cells that were sorted into each bin was used to determine which part of the normal distribution each bin corresponded to, assuming that the leftmost and rightmost expression bins each exclude the most extreme 0.1% of cells. The guide abundance within unsorted cells was quantified and used to estimate guide abundance within the library. A pseudocount was added to each guide count consisting of one read for every 100,000 total reads sequenced in that bin, corresponding to a prior that there is no expression difference for cells containing the guide. For each guide, the mean expression for that guide was estimated by maximizing the likelihood of the observed guide counts for each bin under this model, given that guide’s overall abundance. A *z*-score was estimated for each guide corresponding to how much the mean *TNFAIP3* expression of cells containing that guide differed from those containing non-targeting guides by subtracting the mean of the non-targeting guides.

In order to get element-level statistics, the *z*-scores for each guide were combined in two ways: a significance *z*-score (proportional to a signed *P*-value), and an effect-size *z*-score (the average *z*-score of guides targeting the element). Significance *z*-scores were calculated by applying Stouffer’s method to the individual guide’s *z*-scores. In order to correct these significance *z*-scores for the noise of the assay, they were scaled by the standard deviation of Stouffer *z*-scores calculated from the non-targeting guides. These scaling factors were calculated independently for every number of guides per targeted element *n* (since the noise in the Stouffer *z*-score depends on the number of guides used to calculate it). For example, Stouffer *z*-scores for elements targeted with *n* = 5 guides were normalized by the standard deviation of non-targeting Stouffer *z*-scores, each calculated from randomly sampled groups of five non-targeting guides. Here, non-targeting Stouffer *z*-scores were calculated by sampling the non-targeting guides into groups of size *n*, including each non-targeting guide 10 times total, and calculating a set of Stouffer *z*-scores from each sampling, and using the standard deviation of these *z*-scores to scale the significance *z*-scores for each element for that *n*. *P*-values were then calculated from these *z*-scores, considering only one-tailed tests (downregulation for CRISPRi and upregulation for CRISPRa). For an element to be considered significantly regulating *TNFAIP3*, we required that both replicates’ Benjamini–Hochberg FDRs were less than sqrt(0.1) (i.e. combined FDR < 0.1, and both replicates close to significant independently) and for which the direction of expression change was identical. In cases where there were more than two replicates, we included only the two replicates for which the *TNFAIP3* promoter positive control guides showed the strongest effect. Element- and guide-level data are available in Supplementary Data 5, 6.

### MPRA

MPRA oligosynthesis and cloning was adapted from refs. ^16,71^, tagging each allele with an average of ~250 DNA barcodes. Oligos were synthesized by Agilent Technologies containing 150 bp of genomic context and 15 bp of adapter sequence at either end (5′-ACTGGCCGCTTGACG[150 bp oligo]CACTGCGGCTCCTGC-3′; Supplementary Data 8; 180 bp total). 20 bp barcodes and additional adapter sequences were added by performing 28 emulsion PCR reactions each 50 μL in volume containing 1.86 ng of oligo, 25 μL of Q5 NEBNext MasterMix (NEB, M0541S), 1 unit Q5 HotStart polymerase (NEB, M0493S), 0.5 μM MPRA_v3_F and MPRA_v3_20I_R primers (Supplementary Data 11) and 2 ng BSA (NEB, B9000). PCR master mix was emulsified by vortexing with 220 μL Tegosoft DEC (Evonik), 60 μL ABIL WE (Evonik) and 20 μL mineral oil (Sigma, M5904) per 50 μL PCR reaction at 4 °C for 5 min. 50 μL of Emulsion mixture was added to each well of a 96-well plate and cycled with the following conditions; 95 °C for 30 s, 15 cycles of (95 °C for 20 s, 60 °C for 10 s, 72 °C for 15 s), 72 °C for 5 min. Amplified emulsion mixture was broken and purified by adding 1 mL of 2-butanol (VWR, AA43315-AK), 50 μL of AMPure XP SPRI (Beckman Coulter, A63881) and 80 μL of binding buffer (2.5 M NaCl, 20% PEG-8000) per 350 μL of Emulsion mix and vigorously vortexed followed by incubation for 10 min at room temperature. Broken emulsion/butanol mixture was spun at 2900 × *g* for 5 min and the butanol phase was discarded. The aqueous phase was placed on a magnetic rack for 20 min prior to aspiration. Remaining beads were washed once with 2-butanol, three times with 80% EtOH and eluted in EB (Qiagen, 19086) to yield our barcoded oligo pool.

To create our mpra∆orf library, barcoded oligos were inserted into SfiI digested pMPRA-lenti2 (pMPRA-lenti1∆Sfi1; pMPRA-lenti1: Addgene, 61600) by Gibson Assembly (NEB, E2611) using 1.1 μg of oligos and 1 μg of digested vector in a 40 μL reaction incubated for 60 min at 50 °C followed by AMPure XP SPRI purification and elution in 20 μL of EB. Half of the ligated vector was then transformed into 100 μL of 10-beta e.coli (NEB, C3020K) by electroporation (2 kV, 200 Ω, 25 μF). Electroporated bacteria were immediately split into eight 1 mL aliquots of SOC (NEB, B9020S) and recovered for 1 h at 37 °C then independently expanded in 20 mL of LB supplemented with 100 μg/mL of carbenicillin (EMD, 69101-3) on a floor shaker at 37 °C for 6.5 h. After outgrowth aliquots were pooled prior to plasmid purification (QIAGEN, 12963). For each of the aliquots we plated serial dilutions after SOC recovery and estimated a library size of ~3.2 × 10^6^ CFUs, representing ~250 barcodes per allele.

To insert the *TNFAIP3* promoter and GFP ORF, 20 μg of mpra:∆orf plasmid was linearized with XbaI (NEB, R0145S) and KpnI-HF (NEB, R3142S) and 1x cutsmart buffer (NEB, B7204S) in a 500 μL volume for 3.5 h at 37 °C, followed by SPRI cleaning. An amplicon containing 165 bp of the *TNFAIP3* ORF, GFP open-reading frame and a partial 3′ UTR was then inserted by Gibson assembly using 10 μg of XbaI and KpnI linearized mpra∆orf plasmid, 33 μg of the pTNFAIP3/GFP amplicon in 400 μL of total volume for 90 min at 50 °C followed by a 1.5× beads/sample SPRI purification. The total recovered volume was digested a second time to remove remaining uncut vectors by incubation with KpnI and XbaI in a 100 μL reaction for 6 h at 37 °C followed by Ampure XP purification and elution with 55 μL of Buffer EB.

10 μL of the mpra:pTNFAIP3:gfp plasmid was electroporated (2 kV, 200 Ω, 25 μF) into 220 μL of 10-beta cells. Electroporated bacteria was split across six tubes and each recovered in 2 mL of SOC for 1 h at 37 °C then added to 500 mL of LB with 100 μg/mL of carbenicillin and grown for 9 h at 37 °C prior to plasmid purification (Qiagen, 12991). The plasmid prep was then normalized to 1 μg/μL to generate our final mpra:pTNFAIP3:gfp library used for transfection and lentiviral delivery.

For all transfections, cells were grown to a density of ~1 × 10^6^ cells/mL and 5 × 10^7^ cells were used for each experiment. Cells were collected by centrifugation at 300 × *g* and eluted in 550 μL of RPMI with 55 μg of mpra:pTNFAIP3:gfp library. Electroporation was performed in 100 μL volumes with the Neon transfection system (Life Technologies) applying three pulses of 1200 V for 20 ms each (GM12878) and three pulses of 1325 V for 10 ms each (Jurkat). Using separate control transfections, we achieved transfection efficiencies of 40–60% for all replicates. Cells were allowed to recover in 180 mL in RPMI with 15% FBS for 24 h then collected by centrifugation, washed once with PBS, collected and frozen at −80 °C.

For all transductions, 500 × 10^6^ cells were split into 24-well plates (2M per well in 1 mL of media, 10 plates) infected with lentivirus at an MOI > 1 using polybrene (8 μg/mL) using spin transduction (1760 × *g*, 90 min, 32 °C). Cells were then pooled and centrifuged at 500 × *g*, the viral supernatant was aspirated, and the cells were resuspended in fresh media at 5 × 10^5^ cells/mL, and cultured for 4 days maintaining a density between 2 and 10 × 10^5^/mL. Cells were then harvested through centrifugation at 500 × *g*, washed with PBS, centrifuged again, and cell pellets were frozen at −80 °C.

Total RNA was extracted from cells using Qiagen Maxi RNeasy (Qiagen, 75162) following the manufacturer’s protocol including the on-column DNase digestion. A second DNase treatment was performed on the purified RNA using 5 μL of Turbo DNase (Life Technologies, AM2238) with buffer, in 750 μL of total volume for 1 h at 37 °C. The digestion was stopped with the addition of 7.5 μL 10% SDS and 75 μL of 0.5 M EDTA followed by a 5 min incubation at 70 °C. The total reaction was then used for pulldown of GFP mRNA. Water was added to the DNase digested RNA to bring the total volume to 898 μL to which 900 μL of 20X SSC (Life Technologies, 15557-044), 1800 μL of Formamide (Life Technologies, AM9342) and 2 μL of 100 μM biotin-labeled GFP probe (GFP_BiotinCapture_1-3, IDT, Supplementary Data 11) were added and incubated for 2.5 h at 65 °C. Biotin probes were captured using 400 μL of pre-washed Streptavidin beads (Life Technologies, 65001) eluted in 500 μL of 20X SSC. The hybridized RNA/probe bead mixture was agitated on a nutator at room temperature for 15 min. Beads were captured by magnet and washed once with 1× SSC and twice with 0.1× SSC. Elution of RNA was performed by the addition of 25 μL water and heating of the water/bead mixture for 2 min at 70 °C followed by immediate collection of eluent on a magnet. A second elution was performed by incubating the beads with an additional 25 μL of water at 80 °C. A final DNase treatment was performed in 50 μL total volume using 1 μL of Turbo DNase with addition of the buffer incubated for 60 min at 37 °C followed by inactivation with 1 μL of 10% SDS and purification using RNA clean SPRI beads (Beckman Coulter, A63987).

First-strand cDNA was synthesized from half of the DNase-treated GFP mRNA with SuperScript III and a primer specific to the 3′ UTR (MPRA_v3_Amp2Sc_R, Supplementary Data 11) using the manufacturer’s recommended protocol, modifying the total reaction volume to 40 μL and performing the elongation step at 47 °C for 80 min. Single-stranded cDNA was purified by SPRI and eluted in 30 μL EB.

To minimize amplification bias during the creation of cDNA tag sequencing libraries, samples were amplified by qPCR to estimate relative concentrations of GFP cDNA using 1 μL of sample in a 10 μL PCR reaction containing 5 μL Q5 NEBNext master mix, 1.7 μL Sybr green I diluted 1:10,000 (Life Technologies, S-7567) and 0.5 μM of TruSeq_Universal_Adapter and MPRA_Illumina_GFP_F primers (Supplementary Data 11). Samples were amplified with the following qPCR conditions: 95 °C for 20 s, 40 cycles (95 °C for 20 s, 65 °C for 20 s, 72 °C for 30 s), 72 °C for 2 min. The number of cycles for sample amplification was 1−*n* (the number of cycles it took for each sample to pass the threshold) from the qPCR. To add Illumina sequencing adapters, 10 μL of cDNA samples and mpra:pTNFAIP3:gfp plasmid control (diluted to the qPCR cycle range of the samples) were amplified using the reaction conditions from the qPCR scaled to 50 μL, excluding Sybrgreen I. Amplified cDNA was SPRI purified and eluted in 40 μL of EB. Individual sequencing barcodes were added to each sample by amplifying the entire 40 μL elution in a 100 μL Q5 NEBNext reaction with 0.5 μM of TruSeq_Universal_Adapter primer and a reverse primer containing a unique 8 bp index (Illumina_Multiplex, Supplementary Data 11) for sample demultiplexing post-sequencing. Samples were amplified at 95 °C for 20 s, six cycles (95 °C for 20 s, 64 °C for 30 s, 72 °C for 30 s), 72 °C for 2 min. Indexed libraries were SPRI purified and pooled according to molar estimates from Agilent TapeStation quantifications. Samples were sequenced using 1 × 30 bp chemistry on an Illumina HiSeq 2500 or NextSeq.

To determine oligo/barcode combinations within the MPRA pool, Illumina libraries were prepared from the mpra∆orf plasmid library by performing four separate amplifications with 200 ng of plasmid in a 100 μL Q5 NEBNext PCR reaction containing 0.5 μM of TruSeq_Universal_Adapter and MPRA_v3_TruSeq_Amp2Sa_F primers (Supplementary Data 11) with the following conditions: 95 °C for 20 s, 6 cycles (95 °C for 20 s, 62 °C for 15 s, 72 °C for 30 s), 72 °C for 2 min. Amplified material was SPRI purified using a 0.6× bead/sample ratio and eluted with 30 μL of EB. Sequencing indexes were then attached using 20 μL of the eluted product and the same reaction conditions as for the tag-seq protocol, except the number of enrichment cycles was lowered to 5. Samples were molar pooled and sequenced using 2 × 150 bp chemistry on Illumina HiSeq 2500 and NextSeq.

MPRA RNA output and DNA input sequencing reads were mapped to the known tag sequences using a custom python script (quantifyRNATags.py; available from https://github.com/Carldeboer/MPRAs), allowing for up to four mismatches within the constant region (the common sequence before the tag sequence) and no mismatches within the tag sequence. The barcode counts were input, and tags having fewer than 30 reads in the input (DNA) or 4 reads in the output (RNA) were excluded from subsequent analysis. The log(DNA/RNA) ratio (expression) was calculated using raw counts, scaled so that the median expression is 0, and the expression levels G+C-content normalized such that the mean expression for every %G+C was 0. Finally, to eliminate instances where the tag sequence modifies the apparent expression level, any tags containing any one of eight blackballed 5-mer DNA sequences were excluded. Blackballed 5-mers were defined as those for which the absolute value of the average expression level of all tags containing that 5-mer was >0.15.

SNPs were tested for allele-specific reporter activity by a two-sided Student’s *t*-test, comparing the normalized log(RNA/DNA) expression values for the tags for allele A compared to the tags for allele B. Only SNPs for which we had at least 80 good tags between the two alleles were tested. *P*-values were corrected for multiple hypothesis testing by Benjamini–Hochberg FDR correction. Only SNPs that had an FDR < 0.1 for at least two of the replicates and where the direction of allele-specific reporter activity was consistent between all replicates were considered to be significant.

### Predicted TF-binding perturbation

In order to find TFs whose motifs were disrupted, both alleles were scanned for each SNP with human and selected mouse motifs from CIS-BP^72^ using VEP^56^ and a custom VEP module implementing the GOMER approach^73^ for motif scanning (https://github.com/Carldeboer/VEP_GOMER). In order to be considered a motif disruption, the region surrounding the SNP must both be bound on one or both alleles (>95% of >1% MAF SNPs), and the binding score difference in the log binding score between the alleles must be at least 0.1 (roughly corresponding to about ~1% of SNPs being perturbed per motif). Both code and motifs for this analysis are available here: https://github.com/Carldeboer/VEP_GOMER.

### Data integration and analysis

In order to gauge how much each assay enriched for GWAS signal (as in Fig. 3), we considered all GWAS tag SNPs. Since the set of causal SNPs remains unknown, we must use the set of potentially causal SNPs as an enriched gold standard (e.g. fine mapped variants, or SNPs in tight LD (*r*^2^ > 0.8), as used here). However, a single causal variant could underlie multiple GWAS tag SNPs, for instance, if the causal SNP is in tight LD with both tag SNPs in the GWAS population. Although a single GWAS tag SNP could represent multiple underlying causal SNPs, we expect this to be uncommon, and a potential explanation featuring fewer causal SNPs should be favored. With these considerations in mind, we evaluated each assay for its ability to identify GWAS tag SNPs by being in tight LD to hits. Since some assays could not assay every variant, only assayed variants are included. Similarly, if a tag SNP had no assayed SNPs in tight LD, that tag SNP was not included in evaluation of the assay since it could not have been recovered by the assay. The pseudo-precision and pseudo-recall were calculated for each assay as described in the “Results” section. The enrichment analysis using credible sets instead of tag SNPs was performed identically, but instead of evaluating the recovery of tag SNPs by being in tight LD to hits, we evaluated the recovery of credible sets by having one or more hits within each credible set.

In order to gauge the significance of enrichment for each assay with limited tag SNPs, we created an empirical null distribution by randomizing the data. Since some of the assays (e.g. DHS, CRISPRi/a) have an inherent clustering of their hits (i.e. SNPs within the same enhancer will share the same hit status), our null aimed to preserve this clustering. Specifically, the null was derived by ordering the assayed SNPs by genomic position and reassigning hit status *H*_*a,s*_*’* = *H*_*a,((s+i)* mod *n)*_ for every possible *i* (0 < *i* < *n;* where *n* is the number of assayed SNPs and mod is the modulo operation), and, each time (i.e. for each value of *i*), calculating pseudo-precision and pseudo-recall. *P*-values represent the fraction of this empirical null with at least as high a pseudo-precision and pseudo-recall as that observed from the actual data. We also tested random permutation of the SNP hit status as an alternative to shifting. Here, we used 1000 independent random permutations of the SNP hits to create the null model. Although both approaches yielded similar results (Fig. 3 genomic shifts and Supplementary Fig. 9d, e permutation), we opted to focus on the random null created by shifting hit status; the random permutations fail to capture the clustering of hits that results from genomic proximity and shared hit origins (e.g. adjacent SNPs in the same open chromatin region). For example, if there was only a single functional enhancer with CRISPRi which contained 10 SNPs, and the clustering of these SNPs prioritizes only one GWAS signal in one region, the distribution of these SNPs randomly could result in as many as 10 GWAS positive results in a null permutation test. These null distributions form a straight line in Fig. 3 because the numerator for both is the number of GWAS tag SNPs recovered (*n*_*TH*_) and the denominators for both pseudo-precision and pseudo-recall are invariant across the randomization (*n*_*H*_ for pseudo-precision and *n*_*T*_ for pseudo-recall).

### Reporting summary

Further information on research design is available in the Nature Research Reporting Summary linked to this article.

## Supplementary information

Supplementary Information

Description of Additional Supplementary Files

Supplementary Data 1

Supplementary Data 2

Supplementary Data 3

Supplementary Data 4

Supplementary Data 5

Supplementary Data 6

Supplementary Data 7

Supplementary Data 8

Supplementary Data 9

Supplementary Data 10

Supplementary Data 11

Reporting Summary

## Data Availability

Raw and processed sequencing data for this study are available on NCBI GEO, under accession “GSE136703”. Other sources for data that support our findings are available from: 1000 Genomes, ENCODE, ChIP-Atlas, Immunobase, and GWAS Catalog.

## Code availability

CRISPR analysis software is available at the following link: https://github.com/Carldeboer/MAUDE. Both code and motifs for TF binding motif analysis are available at the following link: https://github.com/Carldeboer/VEP_GOMER. Code for processing MPRA data is available at the following link: https://github.com/Carldeboer/MPRAs.
