# Supplementary material for: Prioritizing disease and trait causal variants at the TNFAIP3 locus using functional and genomic features
Source: Nat Commun. 2020 Mar 6;11:1237. doi: 10.1038/s41467-020-15022-4 (PMC7060350; doi:10.1038/s41467-020-15022-4)
Supplement: Supplementary file 1 — Supplementary Information [file 41467_2020_15022_MOESM1_ESM.docx]

**Prioritizing disease and trait causal variants in the TNFAIP3 locus based on genomic features**

**Ray, et al.**

**Supplementary Information**

Supplementary Figures 1-10

Supplementary References

**
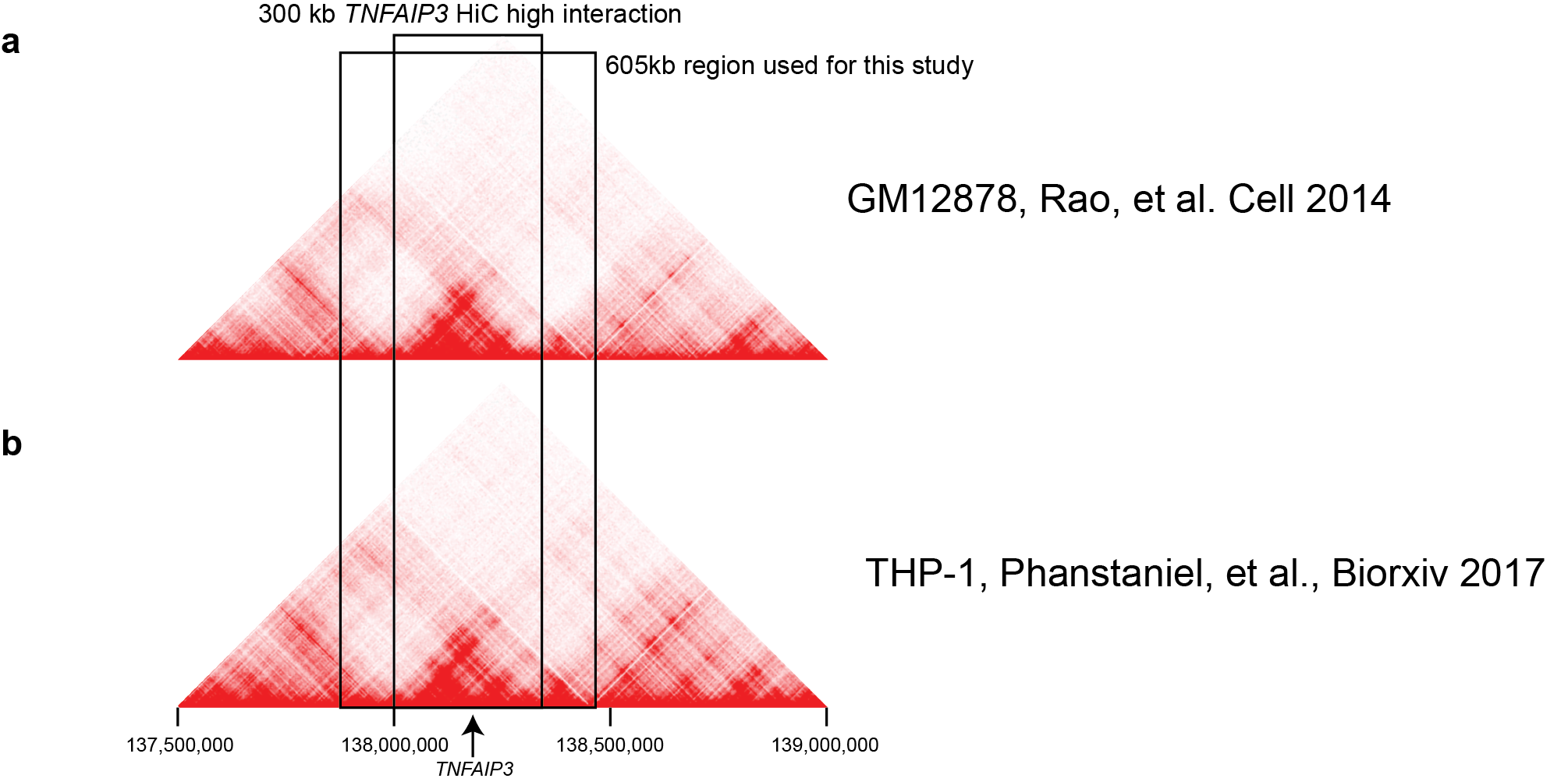
**

**Supplementary Fig. 1.** **Interaction frequency plots for different cell types in the TNFAIP3 locus.** HiC interaction frequency plots for (**a**) GM12878^1^ and (**b**) THP-1 cell lines^2^, with boxes around a 300 kb high interaction domain and the 605 kb region used in this study. The location of *TNFAIP3* and is indicated at the bottom. Redness is proportional to normalized contact frequency.

**
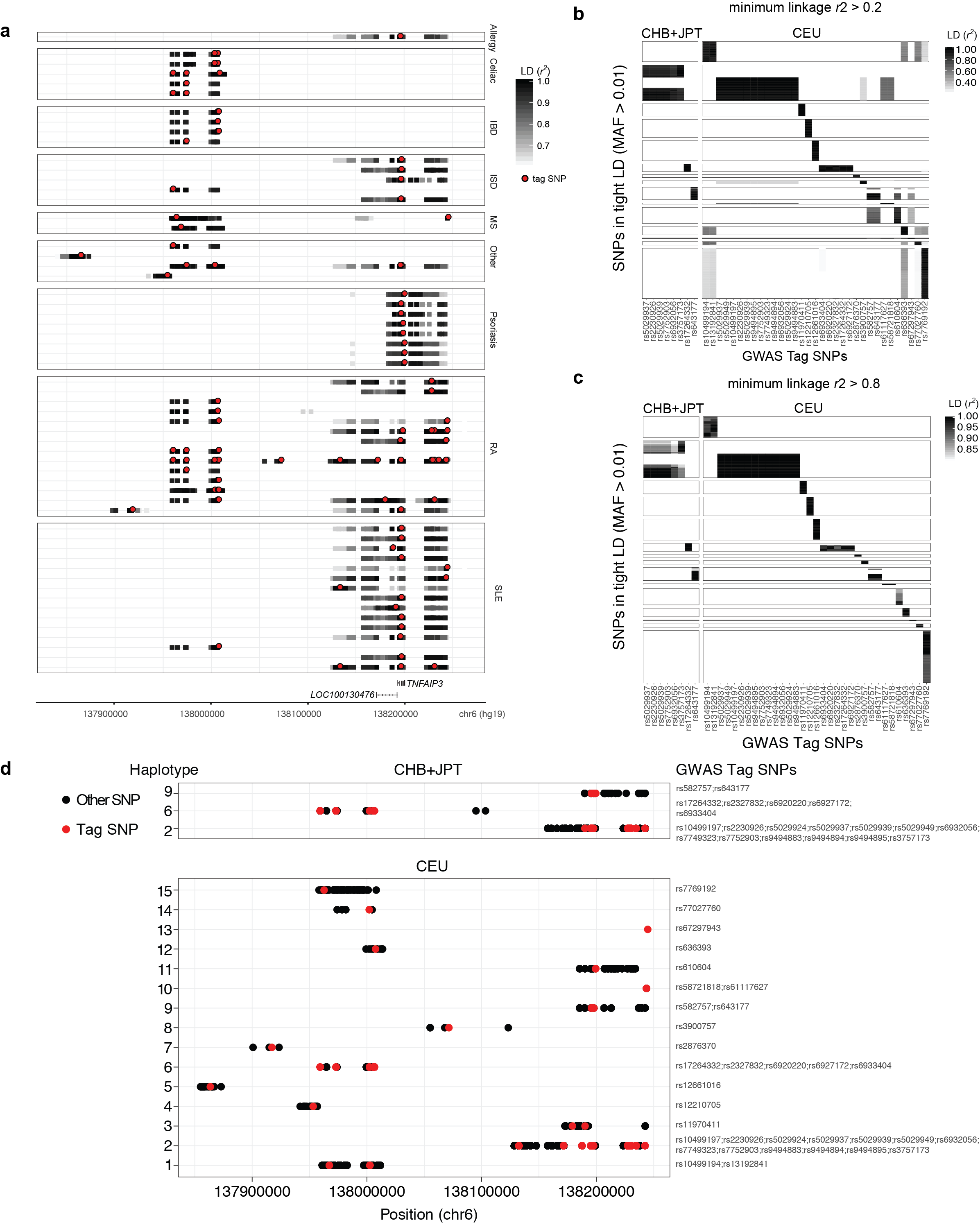
**

**Supplementary Fig. 2. GWAS catalog tag SNPs and haplotypes in the *TNFAIP3* locus.** (**a**) GWAS tag SNPs and variants in tight LD in the *TNFAIP3* locus. Each row represents a study that has significant associations to the *TNFAIP3* locus, grouped by disease type. The *x* axis is the genomic region (hg19), with gene location indicated below. Tag SNPs are marked in red, while SNPs in LD with the tag SNPs are grey bars. The darkness of the bar is proportional to the LD between variants and tag SNPs. (**b**) Heatmap of LD (*r*^2^ > 0.2) between GWAS tag SNPs (*x* axis) and all other variants (*y* axis), grouped by haplotype (vertical boxes). East Asians are shown on the left and Europeans on the right. (**c**) As in (b), but only displaying *r*^2^ > 0.8 (which was used to define disease-associated haplotypes). (**d**) Disease-associated haplotypes. Each haplotype is a row, with the GWAS tag SNPs included in each indicated on the right hand side of the graph. Chromosome 6 position is on the *x* axis, and tag SNPs are indicated in red, with other SNPs on the haplotype in black. Haplotypes associated with disease in East Asians are shown on top, and those in Europeans is shown below.

**
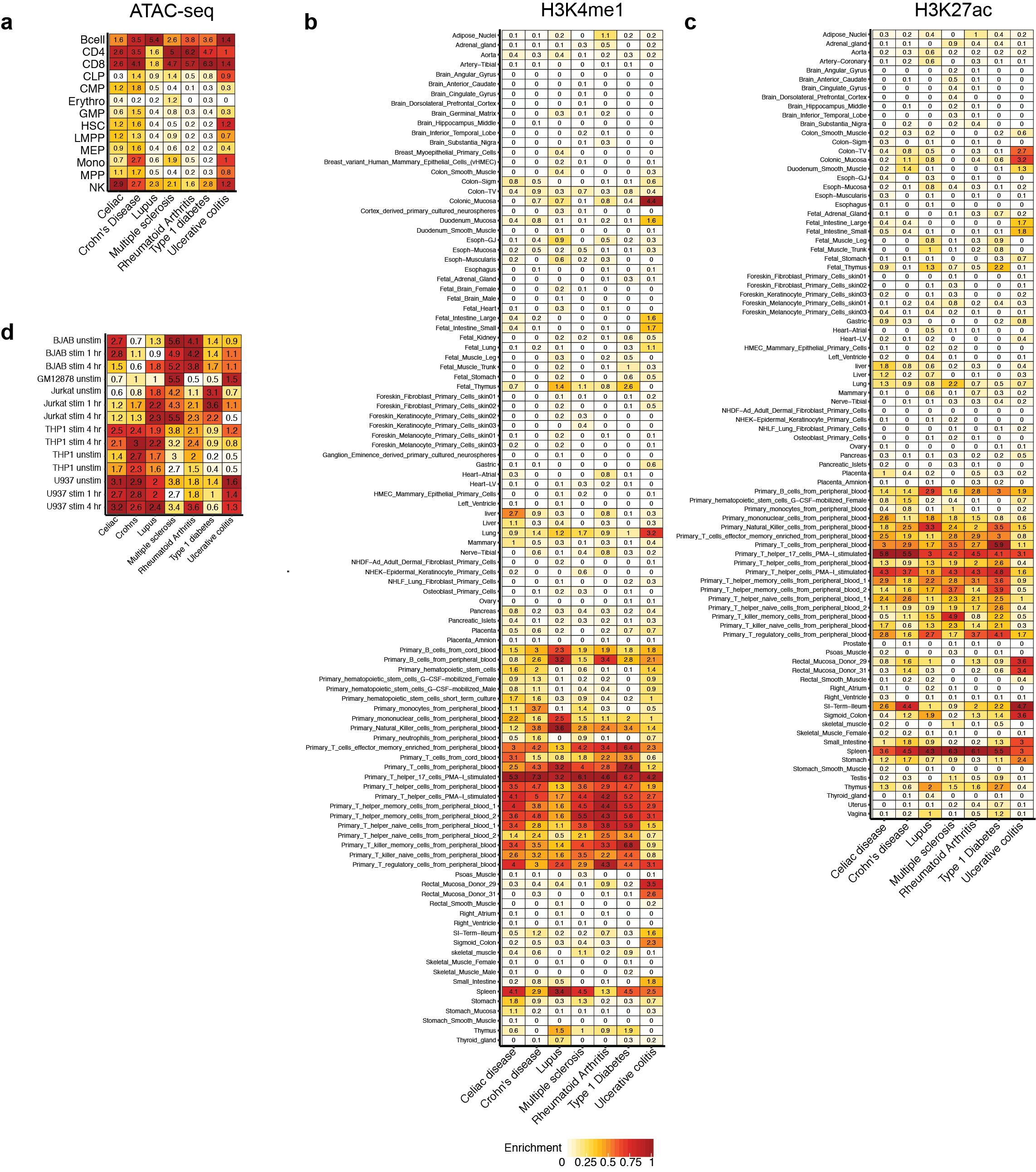
**

**Supplementary Fig. 3. Disease-associated genetic variants are enriched within immune cell-specific accessible and active chromatin regions.**

Enrichment of disease-associated variants in genomic regions associated with (**a**) open chromatin (via ATAC-seq) of the hematopoietic lineage, (**b**) H3K4me1 and (**c**) H3K27ac from ENCODE and Roadmap Epigenomics (http://www.roadmapepigenomics.org/) cell types^3,4^, and (**d**) the cell lines used in this study. In each case, cell types are indicated on the *y* axes, and diseases are indicated on the *x* axes. The number inside each box is the -log10 p-value for the enrichment according to stratified LD score regression. Colour saturation corresponds to proportional enrichment.

**
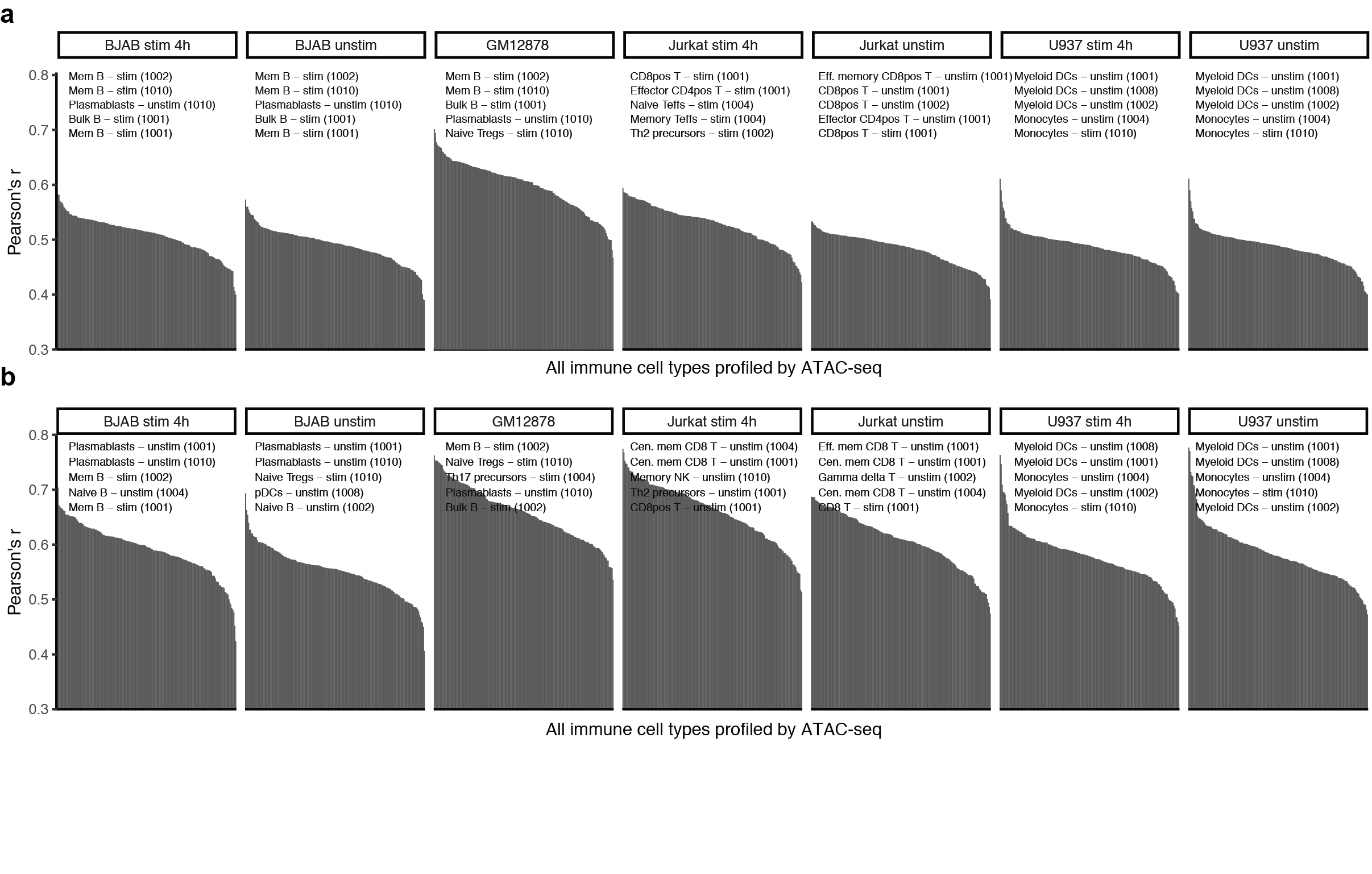
**

**Supplementary Fig. 4. Cell line chromatin accessibility correlates most highly with cell type they were derived from.**

**(a and b)** Pearson correlation coefficient (y axis) of chromatin accessibility profiles between the cell lines used in this study and 32 immune cell types with or without stimulation^5^ for (**a**) the entire genome, and (**b**) the *TNFAIP3* locus. Highlighted are the top 5 correlated cell types from the blood.

**
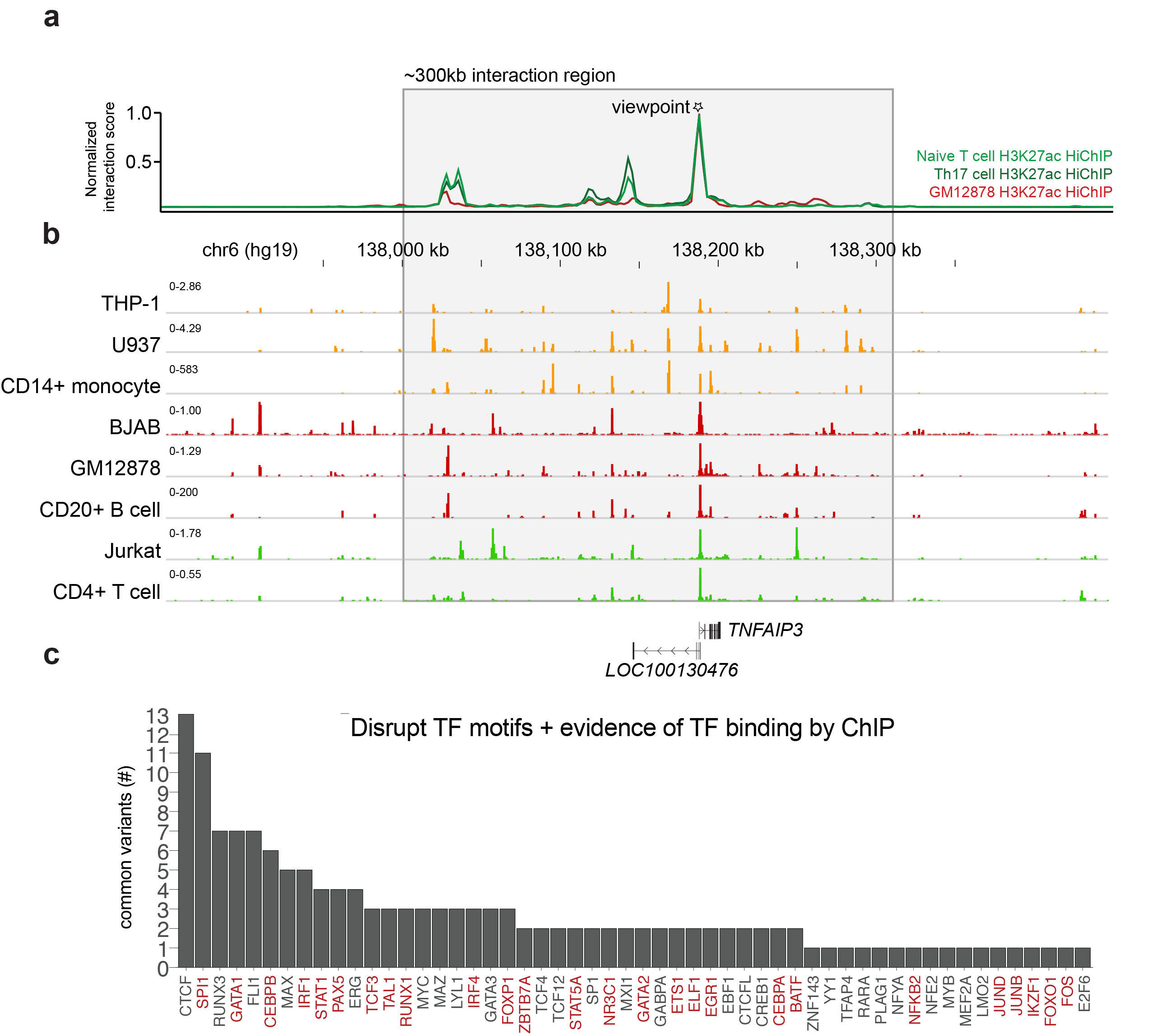
**

**Supplementary Fig. 5. Chromatin state and conformation and TF motif disruption provide context for variant function.**

(**a**) *TNFAIP3* promoter interaction frequency inferred from HiChIP for Th0 and Th17 primary cells, and the GM12878 lymphoblastoid cell line^6^. The *TNFAIP3* promoter (indicated with a star) is used as the viewpoint. (**b**) Accessible chromatin regions surrounding *TNFAIP3* as measured by ATAC-seq for cell types indicated on the left. Genomic location on chromosome 6 is marked on the *x* axis (between a and b), with genes indicated below. (**c**) Putative TF binding disruption by SNPs. Number of SNPs (*y* axis) disrupting each TF (*x* axis), where there is both evidence that the TF binds the region around the SNP (ChIP-seq) and its motif is predicted to be disrupted by the SNP. TF names in red indicate immune-related TFs.

**
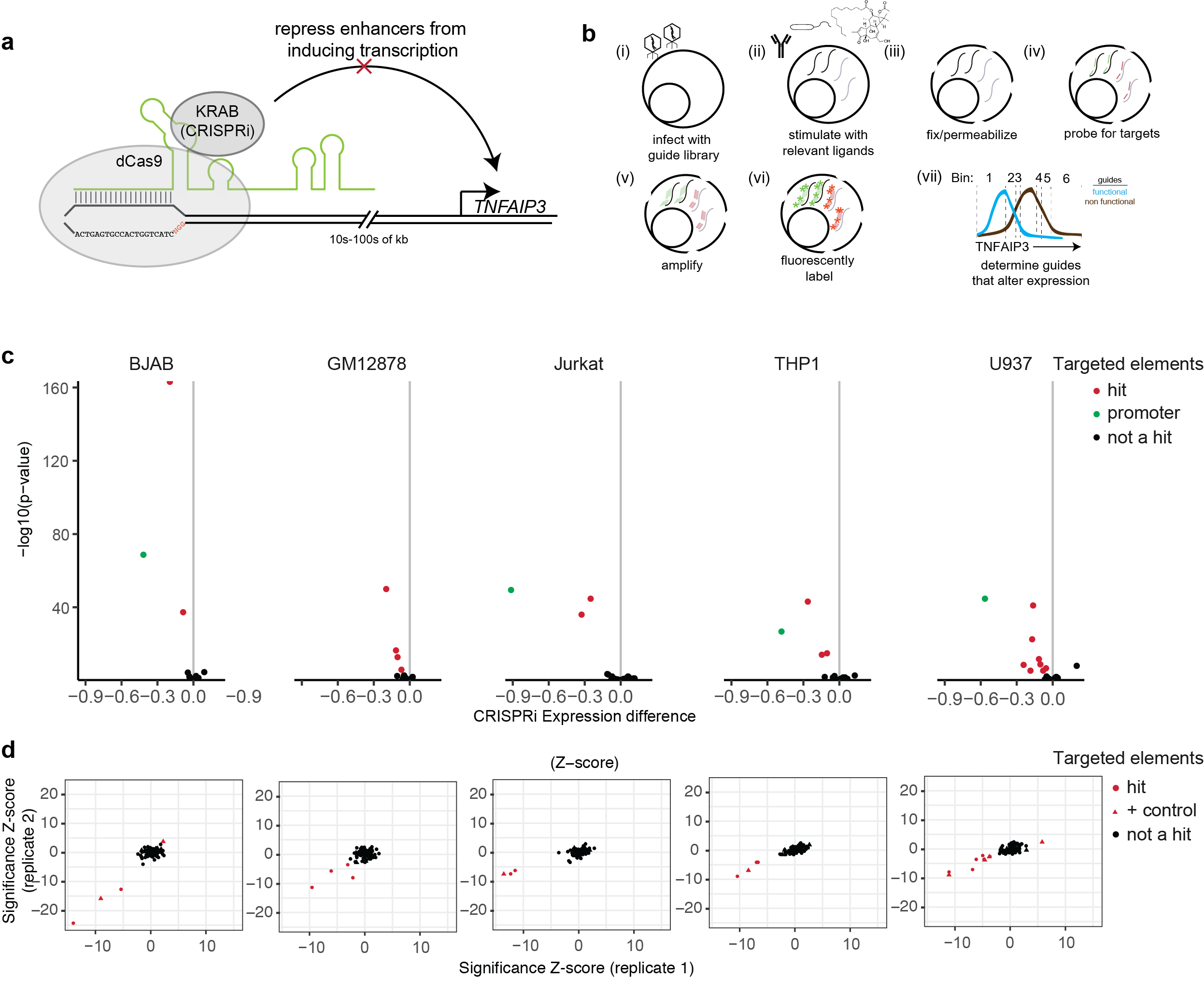
**

**Supplementary Fig. 6. CRISPRi screens identify regions that control TNFAIP3 expression with high reproducibility.**

(**a**) Enhancer repression by CRISPRi. We used CRISPRi targeted to open chromatin to determine which regions, when repressed, lead to a decrease in *TNFAIP3* expression. (**b**) Protocol for FlowFISH. (i) We first infect cell lines that had stable expression of CRISPRi/a with the guide library, (ii) stimulate immune cell lines with the relevant ligands, (iii) fix and permeabilize the cells, (iv) probe for the target transcript, (v) amplify the target signal, (vi) and fluorescently label the amplified probes. (vii) We then sorted cells into six 10% bins on the extremities of the population, and sequenced the bins to determine the prevalence of targeting versus non-targeting guides within each bin. (**c**) Expression changes (*x* axis) by significance (*y* axis) for all CRISPRi targeted regions in all cell lines tested (panels). The region corresponding to the *TNFAIP3* promoter is depicted in green, while other genomic locations around *TNFAIP3* are in black (insignificant) and red (significant). (**d**) CRISPRi results are reproducible. Shown are the significance Z-scores for two replicates (*x* and *y* axes). Circles represent targeted open chromatin regions and positive controls known to positively or negatively regulate *TNFAIP3* are triangles, with red indicating significant *TNFAIP3* regulation and black otherwise. Panels represent cell types as labeled in (c).

**
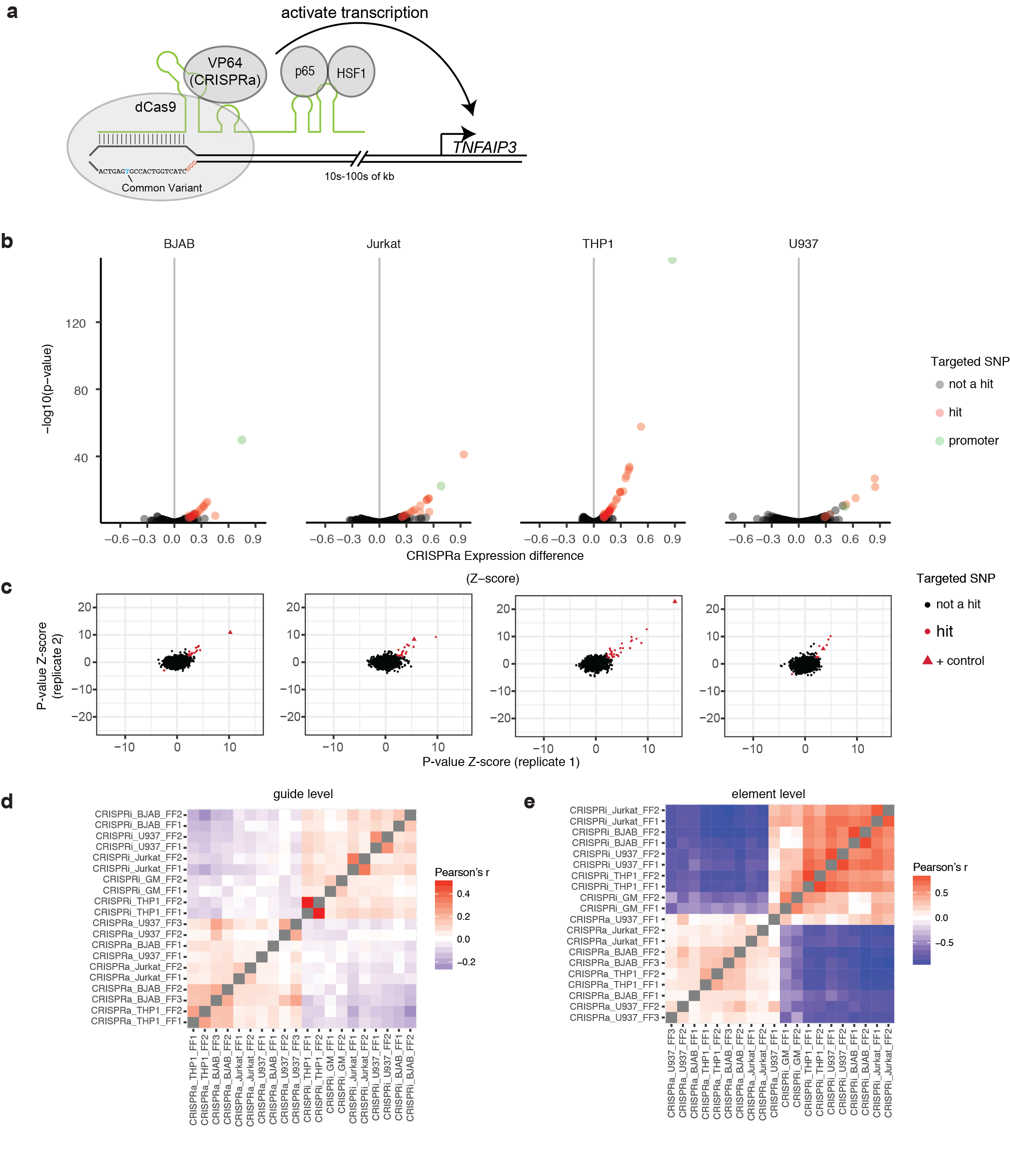
**

**Supplementary Fig. 7. CRISPRa screens map regions that can induce *TNFAIP3* expression with high reproducibility.**

(**a**) Activation of genomic elements by CRISPRa, with which we targeted all variants to determine whether they can function as enhancers of *TNFAIP3* expression. (**b**) Expression changes (*x* axis) by significance (*y* axis) for all CRISPRa targeted regions in all cell lines tested (panels). The region corresponding to the promoter is depicted in green, while other genomic locations around *TNFAIP3* are in black (insignificant) and red (significant). (**c**) CRISPRa replicates are reproducible. Shown are the significance Z-scores for two replicates (*x* and *y* axes). Circles represent each targeted region (within 25 bp of each SNP) and positive controls known to positively or negatively regulate *TNFAIP3* are triangles, with red indicating significant *TNFAIP3* regulation, and black otherwise. Panels represent cell types as labeled in (b). Pairwise correlations between CRISPRi and CRISPRa effect sizes (d) at the guide level and (e) element level, across all replicates (*x* and *y* axes). Colour is proportional to Pearson’s *r*.

**
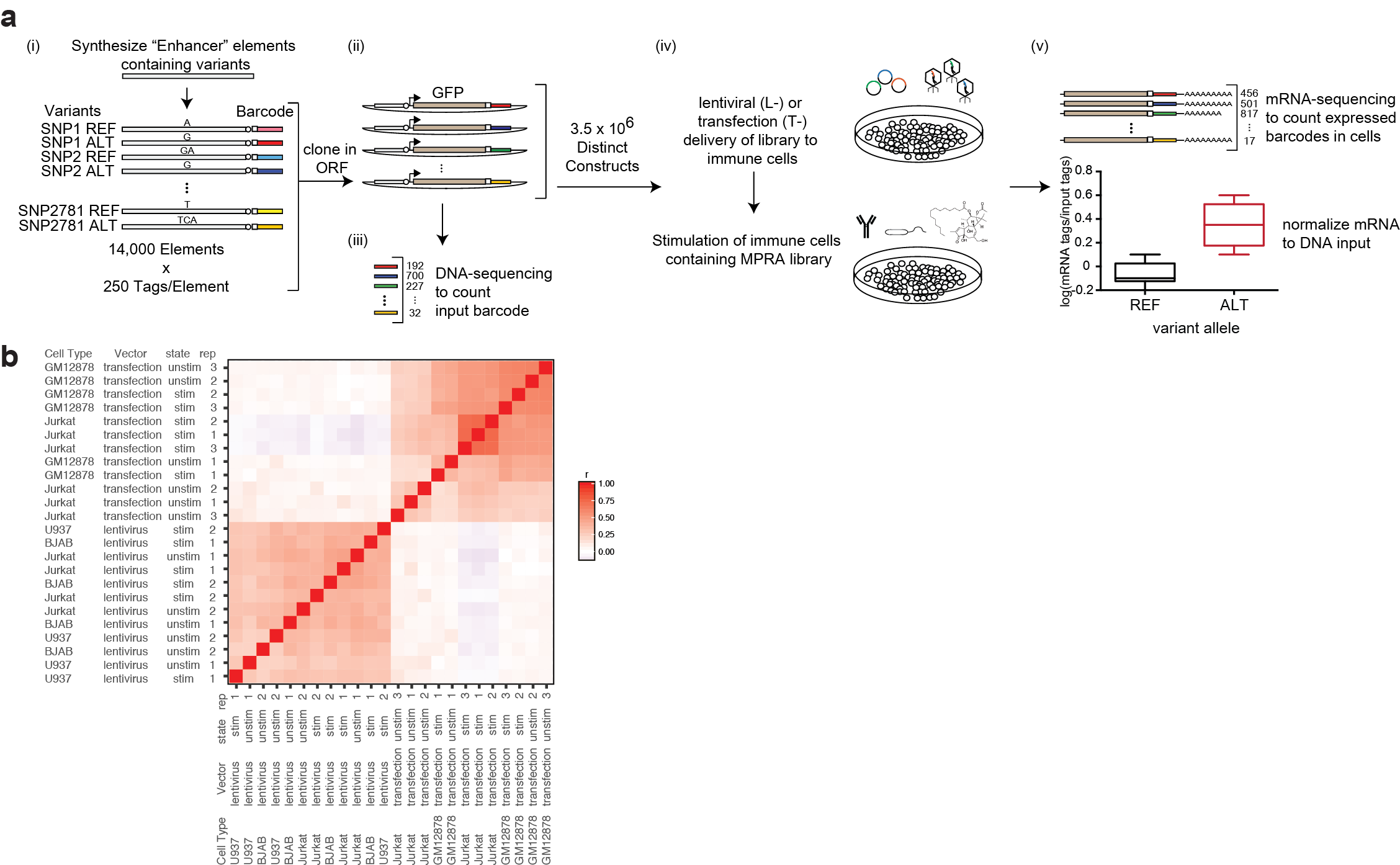
**

**Supplementary Fig. 8. MPRA identifies variants with allele-specific skew, with lentiviral and transfection assays showing markedly different results.**

(**a**) MPRA workflow. (i) Synthesized variants centered in 150 bp of reference DNA are tagged with DNA barcodes, (ii) and then cloned into the MPRA construct. (iii) The library is sequenced to quantify input tag abundances, (iv) and is then either delivered with transfection (T-MPRA) or lentivirus (L-MPRA) to cell lines, which are either left unstimulated or activated with relevant immune ligands. (v) RNA is then harvested and expression of each allele is quantified by RNA-sequencing the expressed barcodes. The RNA barcodes are then normalized to the prevalence of the barcode within the DNA library (from iii), and reference and alternate alleles are compared for their ability to promote reporter expression. (**b**) Pairwise correlation (Pearson’s *r*; colour) between allele-specific expression for all MPRA experiments (all cell types, stimulation conditions, and delivery methods; *x* and *y* axes). Although samples are correlated within each delivery method, they are uncorrelated between lentiviral and transfection delivery.

**
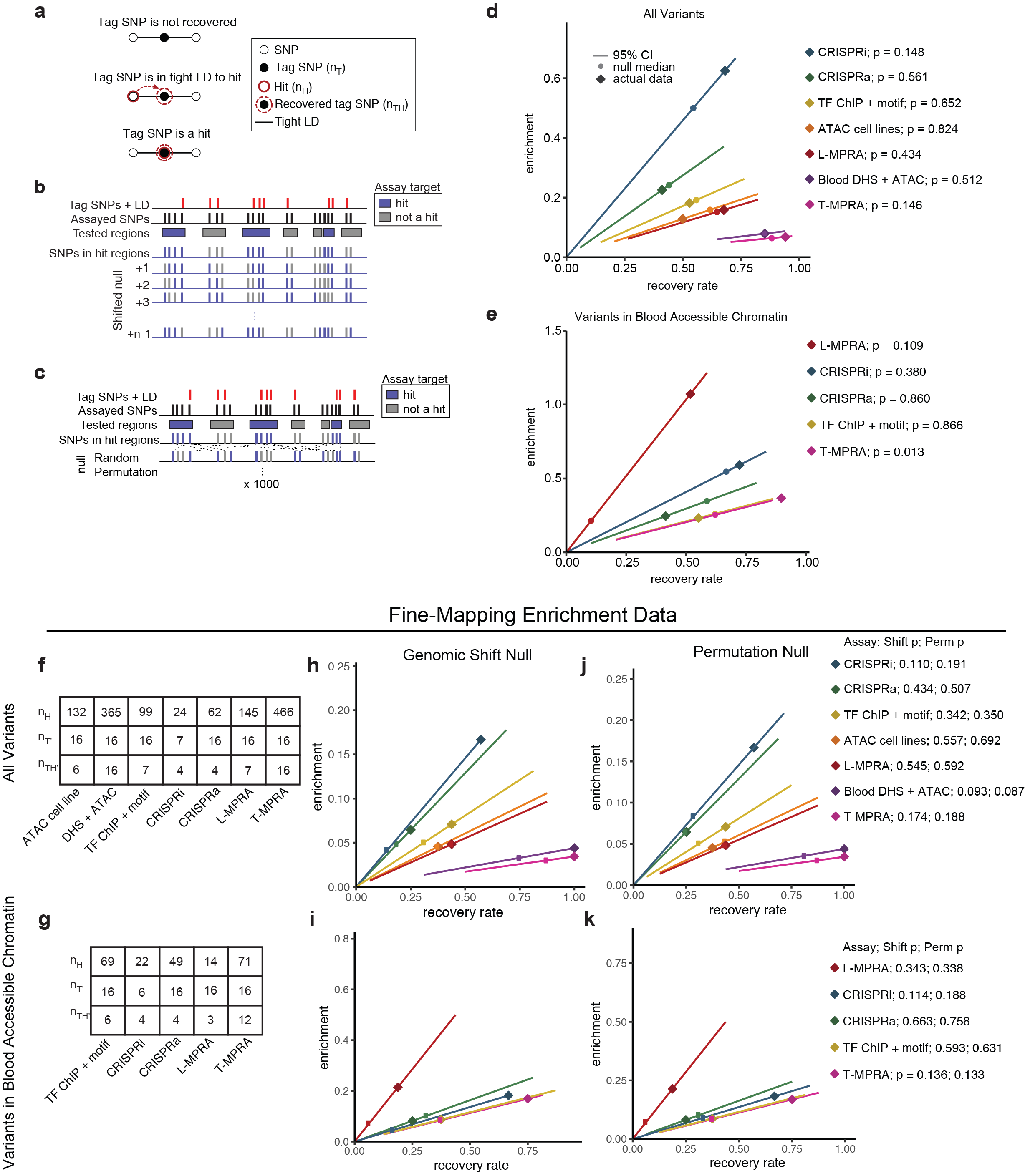
**

**Supplementary Fig. 9.** **GWAS enrichment definition, null models, and fine-mapping enrichment.**

(**a**) GWAS-enrichment definition. For each assay, we calculate the number of SNPs that are assay hits ($n_{H}$), GWAS tag SNPs in tight LD (*r*^2^ > 0.8) with at least one assayed SNP ($n_{T}$), and tag SNPs in tight LD with at least one hit ($n_{TH}$). Pseudo-precision and pseudo-recall are calculated using these values. (**b**) Procedure for creating null hypothesis by genomic shifts. Amongst the subset of SNPs that were assayed, SNPs are arranged by genomic position, and the hit status of each SNP is replaced with that of the upstream SNP from the previous iteration (genomic shifts), with the first SNP replaced with the hit status of the final SNP. This is repeated for all possible shifts, excluding the unshifted (actual) data, and each time, pseudo-precision and pseudo-recall are calculated as in (a) to create the null distribution (**c**) Procedure for creating a null model by permutation. Amongst the subset of SNPs that were assayed, each SNP is randomly assigned a hit status at each iteration of the permutation, each time preserving the total number of hits. With each permutation, pseudo-precision and pseudo-recall are calculated as in **Fig. 3a** to create the null distribution. (**d** and **e**) Pseudo-precision (*y* axes) and pseudo-recall (*x* axes) for GWAS enrichment (left) for each assay (colours), with diamonds depicting the actual assay performance, and the lines depicting each assay’s *permuted* null distribution (as in **a**). Empirical one-sided *P*-values derived from the permuted null are indicated next to each assay label. *P*-values are not corrected for multiple hypothesis testing. (**f** and **g**) Values for $n_{H}$, $n_{T'}$, and $n_{TH'}$ for all methods, considering (**f**) all fine-mapped variants, and (**g**) only fine-mapped variants in open chromatin. (**h**-**k**) Pseudo-precision (*y* axes) and pseudo-recall (*x* axes) for GWAS enrichment for each assay (colours), with diamonds depicting the actual assay performance, and the lines depicting the 95% CI of each assay’s null distribution (**h** and **i** are genomic shifts; **j** and **k** are permutation). Empirical one-sided *P*-values derived from each null are indicated next to each assay label. *P*-values are not corrected for multiple hypothesis testing. (**h and j**) Each assay evaluated individually for all tested variants and (**i and k**) considering only SNPs in blood cell accessible chromatin.

**
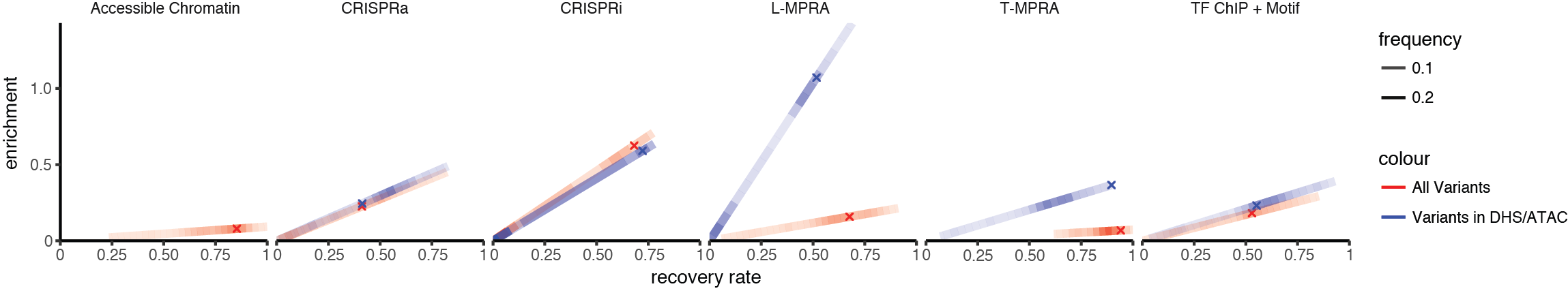
**

**Supplementary Fig. 10.** **Chromatin accessibility increases the pseudo-precision of MPRA but not for other assays.**

Pseudo-precision (*y* axis) and pseudo-recall (*x* axis) for each assay for all variants included in each assay (red) and when considering only variants in accessible chromatin (blue). Colored lines represent the probability density function of an empirically derived random null distribution (shifting null) for each assay (**Methods**), and each X is the actual data.

**Supplementary References**

1 Rao, S. S. P. *et al.* A 3D map of the human genome at kilobase resolution reveals principles of chromatin looping. *Cell* **159**, 1665-1680, doi:10.1016/j.cell.2014.11.021 (2014).

2 Phanstiel, D. H. *et al.* Static and Dynamic DNA Loops form AP-1-Bound Activation Hubs during Macrophage Development. *Molecular Cell* **67**, 1037-1048.e1036, doi:10.1016/j.molcel.2017.08.006 (2017).

3 Consortium, T. E. P. *et al.* An integrated encyclopedia of DNA elements in the human genome. *Nature* **488**, 57-74, doi:10.1038/nature11247 (2012).

4 Corces, M. R. *et al.* Lineage-specific and single-cell chromatin accessibility charts human hematopoiesis and leukemia evolution. *Nature Genetics* **48**, 1193-1203, doi:10.1038/ng.3646 (2016).

5 Calderon, D. *et al.* Landscape of stimulation-responsive chromatin across diverse human immune cells. *Nature Genetics* **51**, 1494-1505, doi:10.1038/s41588-019-0505-9 (2019).

6 Mumbach, M. R. *et al.* HiChIP: efficient and sensitive analysis of protein-directed genome architecture. *Nature Methods* **13**, 919-922, doi:10.1038/nmeth.3999 (2016).
