# Supplementary material for: Prioritizing disease and trait causal variants at the TNFAIP3 locus using functional and genomic features
Source: Nat Commun. 2020 Mar 6;11:1237. doi: 10.1038/s41467-020-15022-4 (PMC7060350; doi:10.1038/s41467-020-15022-4)
Supplement: Supplementary file 2 — Description of Additional Supplementary Files [file 41467_2020_15022_MOESM2_ESM.pdf]

Title: Supplementary Data 1.

Description: A summary of the GWAS tag SNPs and trait associations with disease-associated haplotypes.

Title: Supplementary Data 2.

Description: Fine-mapping using FINEMAP and SuSiE. Credible sets in the *TNFAIP3* locus for UK Biobank and Biobank Japan immune-related phenotypes

Title: Supplementary Data 3.

Description: Collective functional data for all TNFAIP3 locus common variants.

Table of variants complete with all functional data annotating each variant.

Title: Supplementary Data 4.

Description: CRISPRi screen oligos. Oligonucleotides synthesized for CRISPRi screens.

Title: Supplementary Data 5.

Description: Element-level CRISPRi and CRISPRa scores and statistics. Table of effect sizes, significance *z*-scores, one-sided MAUDE *P*-values, and FDRs for all replicates and cell types on the element level for CRISPRi and CRISPRa screens.

Title: Supplementary Data 6.

Description: Guide-level CRISPRi and CRISPRa Z-scores. Table of guide-level Z-scores for all replicates and cell types for CRISPRi and CRISPRa screens.

Title: Supplementary Data 7.

Description: CRISPRa screen oligos. Oligonucleotides synthesized for CRISPRa screens.

Title: Supplementary Data 8.

Description: MPRA oligos. Oligonucleotides synthesized for MPRA screens.

Title: Supplementary Data 9.

Description: MPRA variant scores and statistics. Table of uncorrected two-sided *t*-test *P*-values comparing the barcode expression between the two alleles, allelic skews, and Q values (Benjamini-Hochberg multiple hypothesis testing-corrected FDR) for all replicates, cell types, delivery methods, and stimulation conditions for MPRAs.

Title: Supplementary Data 10.

Description: Credible sets from fine-mapping studies. 16 95% credible sets from fine-mapping studies in the *TNFAIP3* locus.

Title: Supplementary Data 11.

Description: Table of Primers. Primers used throughout manuscript.
