# Supplementary material for: Prioritizing disease and trait causal variants at the TNFAIP3 locus using functional and genomic features
Source: Nat Commun. 2020 Mar 6;11:1237. doi: 10.1038/s41467-020-15022-4 (PMC7060350; doi:10.1038/s41467-020-15022-4)
Supplement: Supplementary file 14 — Reporting Summary [file 41467_2020_15022_MOESM14_ESM.pdf]

nature research

Corresponding author(s): Hacohen N, Regev A

Last updated by author(s): 02/11/2020

Reporting Summary

Nature Research wishes to improve the reproducibility of the work that we publish. This form provides structure for consistency and transparency in reporting. For further information on Nature Research policies, see Authors & Referees and the Editorial Policy Checklist .

Statistics

For all statistical analyses, confirm that the following items are present in the figure legend, table legend, main text, or Methods section.

n/a	Confirmed	
x		The exact sample size (*n*) for each experimental group/condition, given as a discrete number and unit of measurement
	x	A statement on whether measurements were taken from distinct samples or whether the same sample was measured repeatedly
	x	The statistical test(s) used AND whether they are one- or two-sided<br>*Only common tests should be described solely by name; describe more complex techniques in the Methods section.*
x		A description of all covariates tested
	x	A description of any assumptions or corrections, such as tests of normality and adjustment for multiple comparisons
x		A full description of the statistical parameters including central tendency (e.g. means) or other basic estimates (e.g. regression coefficient) AND variation (e.g. standard deviation) or associated estimates of uncertainty (e.g. confidence intervals)
	x	For null hypothesis testing, the test statistic (e.g. *F*, *t*, *r*) with confidence intervals, effect sizes, degrees of freedom and *P* value noted<br>*Give P values as exact values whenever suitable.*
x		For Bayesian analysis, information on the choice of priors and Markov chain Monte Carlo settings
x		For hierarchical and complex designs, identification of the appropriate level for tests and full reporting of outcomes
	x	Estimates of effect sizes (e.g. Cohen's *d*, Pearson's *r*), indicating how they were calculated

Our web collection on statistics for biologists contains articles on many of the points above.

Software and code

Policy information about availability of computer code

Data collection	No software was used for data acquisition.
Data analysis	Bowtie (0.12.7), Tabix(0.2.5), hichipper (0.7.2), SAIGE (0.29.4), BOLT-LMM (2.3.2), FINEMAP (1.3), susieR (0.7.1), LDstore (2.0b), vcftools (0.1.15), plink (v1.90b3d), Bowtie2 (2.2.1), Pyatac (0.3.4), Picard (2.20.6), HOMER (4.6), R (3.3.1), Python (2.7.9), MAUDE (https://github.com/Carldeboer/MAUDE), VEP_GOMER (https://github.com/Carldeboer/VEP_GOMER), were used for data analysis.

For manuscripts utilizing custom algorithms or software that are central to the research but not yet described in published literature, software must be made available to editors/reviewers. We strongly encourage code deposition in a community repository (e.g. GitHub). See the Nature Research guidelines for submitting code & software for further information.

Data

Policy information about availability of data

All manuscripts must include a data availability statement. This statement should provide the following information, where applicable:

- Accession codes, unique identifiers, or web links for publicly available datasets
- A list of figures that have associated raw data
- A description of any restrictions on data availability

Sequence data that support the findings are deposited on NCBI GEO: GSE136703
Other sources for data that support are findings are available from:
1000 Genomes: https://www.internationalgenome.org/
ENCODE: https://www.encodeproject.org/
ChIP-Atlas: https://chip-atlas.org/
Immunobase: https://genetics.opentargets.org/immunobase
GWAS Catalog: https://www.ebi.ac.uk/gwas/

Field-specific reporting

Please select the one below that is the best fit for your research. If you are not sure, read the appropriate sections before making your selection.

☒ Life sciences ☐ Behavioural & social sciences ☐ Ecological, evolutionary & environmental sciences

For a reference copy of the document with all sections, see nature.com/documents/nr-reporting-summary-flat.pdf

Life sciences study design

All studies must disclose on these points even when the disclosure is negative.

Sample size	Not applicable; our study does not conduct statistics in populations
Data exclusions	For the CRISPRi/a analysis, we had 2 or 3 replicates. To simplify analysis, in cases where we had three replicates, only the two replicates showing the largest effect when targeting the TNFAIP3 promoter were used, as the guides targeting the promoter were used as a positive controls to assess the efficacy of the CRISPRi/CRISPRa assay. While all replicates were successful, some replicates worked better than others. This was not established prior to our analysis.
Replication	We had 2-3 replicates for every assay, with the exception of ATAC-seq, where we had one replicate per condition and a time course of stimulation. All replicates for each assay were successful.
Randomization	Our analyses did not entail randomization, because most assays occurred in pools, which are internally controlled- we used other statistical methods to obtain true positive results (e.g. establishing a null distribution of known negative controls)
Blinding	Our analyses did not entail blinding, because most assays occurred in pools, which are internally controlled; we used other statistical methods to obtain true positive results (e.g. establishing a null distribution of known negative controls)

Reporting for specific materials, systems and methods

We require information from authors about some types of materials, experimental systems and methods used in many studies. Here, indicate whether each material, system or method listed is relevant to your study. If you are not sure if a list item applies to your research, read the appropriate section before selecting a response.

Materials & experimental systems

n/a	Involved in the study	
☐	☒	Antibodies
☐	☒	Eukaryotic cell lines
☒	☐	Palaeontology
☒	☐	Animals and other organisms
☒	☐	Human research participants
☒	☐	Clinical data

Methods

n/a	Involved in the study	
☒	☐	ChIP-seq
☒	☐	Flow cytometry
☒	☐	MRI-based neuroimaging

Antibodies

Antibodies used	anti-CD3 (OKT3), anti-IgM, anti-CD40

Validation	For anti-CD3 (clone OKT3; Biolegend, 317304), each lot of the antibody is quality tested by the manufacturer through immunofluorescence staining and flow cytometry analysis. Use of this antibody clone for activating CD3 is well established, including in these publications (PMID 3926880, PMID 6609822, PMID 11086054, and others).<br>For anti-IgM (Sigma-Aldrich, 86620270), identity and purity of the antibody is established by the manufacturer through immunoelectrophoresis (IEP). Electrophoresis of the antibody preparation followed by diffusion versus antigoat IgG and anti-goat whole serum result in single arcs of precipitation. Use of this antibody for activating IgM is well established, including in these publications (PMID 18048365, PMID 28566383, and others).<br>For anti-CD40 (ThermoFisher, 14-0409-82), this antibody was previously used for mass cytometry (PMID 28475899), and flow cytometry (PMID 20479117, PMID 24292363, PMID 22323450, and others). Schlossman, et al. 1995 (ed. Leukocyte Typing V:White Cell Differentiation Antigens) first reported on the use of this clone of anti-CD40 for costimulation in B cell proliferation.

Eukaryotic cell lines

Policy information about cell lines

Cell line source(s)	ATCC (Jurkat, U937, THP-1), DSMZ (BJAB), and Coriell (GM12878)
Authentication	none of the cell lines used were authenticated
Mycoplasma contamination	cell lines tested negative for mycoplasma
Commonly misidentified lines<br>(See ICLAC register)	No commonly misidentified lines were used in the study
